# GALNT1 Enhances Malignant Phenotype of Gastric Cancer via Modulating CD44 Glycosylation to Activate the Wnt/β-catenin Signaling Pathway

**DOI:** 10.7150/ijbs.73431

**Published:** 2022-10-17

**Authors:** Junchang Zhang, Han Wang, Jing Wu, Cheng Yuan, Songyao Chen, Shuhao Liu, Mingyu Huo, Changhua Zhang, Yulong He

**Affiliations:** 1 Digestive Disease Center, The Seventh Affiliated Hospital of Sun Yat-Sen University, Shenzhen, Guangdong 518107, P.R. China.; 2 Department of Gastrointestinopancreatic Surgery, The First Affiliated Hospital of Sun Yat-Sen University, Guangzhou, Guangdong 510080, P.R. China.

**Keywords:** Gastric cancer, *GALNT1*, CD44, O-Glycosylation, Wnt/β-catenin signaling pathway

## Abstract

O-glycosylation is a widespread post-translational modification of proteins. Aberrant O-glycosylation is a hallmark of cancer. Here, we show that the polypeptide N-acetylgalactosamine-transferase 1 (*GALNT1*) is frequently upregulated in gastric cancer and is correlated with poor survival. Overexpression of *GALNT1* promoted, whereas knockdown suppressed proliferation, migration, and invasion of gastric cancer cells *in vitro* and *in vivo*. Mechanistically, *GALNT1* enhances aberrant initiation of O-glycosylation and results in CD44 glycoproteins modified with abundant Tn antigens, thereby activating the Wnt/β-catenin signaling pathway. Collectively, this study demonstrates that *GALNT1* overexpression in gastric cancer promotes the Wnt/β-catenin signaling pathway via abnormal O-glycosylation of CD44 to enhance malignancy, providing a novel strategy for the development of therapeutic reagents against gastric cancer.

## Introduction

Gastric cancer (GC) is the fifth most frequent tumor and the fourth leading cause of cancer-related mortality worldwide, despite its decreasing incidence over the past 50 years 1. Although there have been considerable improvements in diagnosis and therapy for GC, metastasis remains the primary cause of death in patients with GC 2, rendering prognosis challenging. Invasive tumor phenotypes are often associated with metastasis and poor survival. Therefore, elucidating the mechanisms that drive the aggressive phenotype of malignant GC progression is vital.

Glycosylation, one of the most prevalent post-translational modifications in proteins, is frequently altered in tumors and associated with tumor progression 3, 4. A frequent and noticeable characteristic of malignancy is the abundance of shortened O-glycans, such as the Tn antigen, which can be recognized by vicia villosa (VVA) lectins and is formed by aberrant initiation of N-acetylgalactosamine (GalNAc)-type O-glycosylation 5, 6. Accumulated studies indicate that the Tn antigen is abnormally increased in human cancers, including breast cancer, lung cancer, and GC tissues 7-9, which is strongly correlated with cancer progression characteristics, such as prognosis, invasion, metastasis, and immune escape 10, 11. The above studies indicate that O- glycosylation is intimately associated with the emergence and progression of cancers. Hence, understanding the involvement of O-glycosylation in GC may open new avenues for elucidating the pathogenesis of gastric cancer.

Polypeptide N-acetylgalactosamine-transferases (GALNTs) are abnormally expressed in multiple cancers and affect tumor progression by initiating GalNAc-type O-glycosylation of various proteins 12-15. Moreover, the initiation of O-glycosylation had previously been demonstrated to begin in the Golgi apparatus 16. In contrast, the initiation of O-glycosylation is associated with GALNTs relocated from the Golgi apparatus to the endoplasmic reticulum (ER) in tumor progression and metastasis (GALNTs activation [GALA] pathway) 8, 17, 18. *GALNT1*, as a member of the GALNT family, initiates mucin-type O-glycosylation by transferring α-GalNAc from UDP-GalNAc to serine (Ser) or threonine (Thr) residues in proteins 11, 19. Importantly, *GALNT1* plays a vital role in cancer growth and metastasis by modifying the O-glycosylation of various glycoproteins, such as mucin 1 (MUC1), osteopontin (OPN), matrix metalloproteinase-14 (MMP14), and integrin α3 17, 18, 20. These reports suggest that *GALNT1* may be an efficient prognostic biomarker and a therapeutic target for treating a variety of malignancies. However, the function and detailed molecular mechanisms of *GALNT1* remain largely unknown.

Therefore, the goal of this study was to explore the underlying mechanism by which *GALNT1* controls GC progression and assess its therapeutic potential.

## Materials and methods

### Clinical data and tissue samples

At the First Affiliated Hospital of Sun Yat‐sen University, tissue samples from 150 patients with GC with detailed medical records were collected between January 2010 and December 2013. Twenty-three fresh GC samples and their neighboring normal samples were collected at the Seventh Affiliated Hospital of Sun Yat-sen University. Total RNA and total protein were extracted from 13 and 10 pairs of tissue samples, respectively. Informed consent was obtained from patients involved in the study. This study was approved by the Ethics Committees of the First and Seventh Affiliated Hospitals of Sun Yat-sen University.

### Cell culture

We used one gastric epithelial cell line (GES1) and eight GC cell lines (AGS, MGC803, KATO III, BGC803, MKN28, SNU719, HGC27 and SGC7901) obtained from the Chinese Academy of Sciences (Shanghai, China). AGS cells were cultured in DMEM/F12 (Gibco, CAT# C11330500BT, Beijing, China) and 10% fetal bovine serum (Gibco, CAT# 10099141C, Beijing, China). Other cells were cultivated in RPMI‐1640 (Gibco, CAT# C11875500BT, Beijing, China) with 10% fetal bovine serum. All the cells were maintained at 37 °C in a 5% CO_2_ cell culture incubator.

### Cell transfection and lentiviral infection

GeneChem (Shanghai, China) provided lentiviral vectors for *GALNT1* knockdown (*shGALNT1*), whereas those for *CD44* knockdown (*shCD44*) were purchased from GeneCopoeia (Guangzhou, China) (Table S1). The *GALNT1* expressing lentiviral vector was constructed by cloning *GALNT1* into the GV341 vector (GeneChem, Shanghai, China). Lentiviral transduction was generated and purified according to the manufacturer's instructions. Puromycin (2 μg/ml) was added to screen the transgenic cells.

### Quantitative real-time PCR

Total RNA from GC cells and fresh tissues was extracted with AG RNAex Pro RNA reagent (Accurate Biology, CAT#AG21102, Hunan, China) according to the manufacturer's instructions. RNA was reverse transcribed to cDNA using the Evo M‐MLV reverse transcription master mix (Accurate Biology, CAT#AG11706, Hunan, China). Quantitative real-time PCR (qRT-PCR) was performing using a SYBR Green Pro Taq HS premixed qPCR kit (Accurate Biology, CAT# AG11702, Hunan, China). Primer sequences are listed in Supplementary Table S2. The 2^-ΔΔCT^ technique was used to analyze the data.

### Western blotting and antibodies

Protein lysis buffer was used to extract total proteins from GC cells and fresh tissues on ice for 30 min in accordance with the procedure. A nuclear and cytoplasmic protein extraction kit (Beyotime Biotechnology, CAT#P0027, Shanghai, China) was used to fractionate the nuclear and cytoplasmic fractions as per the manufacturer's instructions. Protein concentrations were quantified using the BCA protein assay kit (Biosharp, CAT# BL521A). Equal quantities of proteins were fractionated by 10% SDS-PAGE and transferred to PVDF or NC membrane. The transferred protein was blotted with the corresponding antibody overnight at 4 °C after blocking with 5% skim milk for 1 h. The following primary antibodies were used: anti-*GALNT1* (1:500, Abcam, CAT#ab253025), anti-GAPDH (1:2000, Proteintech, CAT#10494-1-AP), anti-CD44 (1:2000, Proteintech, CAT# 15675-1-AP-100), anti-β-catenin (1:5000, Proteintech, CAT#51067-2-AP), anti-phospho-β-catenin (1:1000, Cell Signaling Technology, CAT#4176S), anti-c-Myc (1:2000, Proteintech, CAT#10828-1-AP), anti-Lamin B1(1:1000, Cell Signaling Technology, CAT#13435), and VVA (2 μg/ml, Vector Laboratories, CAT#B-1235-2). HRP‐conjugated anti‐rabbit IgG (1:1000, Beyotime, CAT#A0208), anti‐mouse IgG (1:1000, Beyotime, CAT#A0216), and Streptavidin HRP (1:10000, Abcam, CAT#ab7403-500) were used as the secondary antibodies. Proteins were detected using an extremely sensitive ECL assay kit (Meilunbio, CAT#MA0186-2).

### Immunohistochemistry

Immunohistochemistry (IHC) was performed according to a standard protocol. Tumor tissue sections were deparaffinized in xylol and rehydrated with a stepwise decrease in ethanol concentration. The sections were then heated in a pressure cooker for antigen retrieval with citric acid buffer and cooled naturally to room temperature. After washing with distilled water, the tissue was treated with 3.0% hydrogen peroxide for 15 min at room temperature, followed by 30 min incubation with 5% goat serum. After washing thrice with phosphate-buffered saline (PBS), rabbit anti-*GALNT1* (1:200, Abcam, CAT# ab253025) was used to incubate the sections overnight at 4 °C. Subsequently, the sections were incubated with horseradish peroxidase (HRP) (goat anti-rabbit/mouse, CAT#A0208, Beyotime) for one hour at room temperature after washing in PBS containing Tween-20 (PBST). After 3,3'-diaminobenzidine (DAB) and hematoxylin staining, *GALNT1* expression was evaluated using an inverted microscope. VVA expression in GC tissues was also detected using IHC as described above. Non-specific binding was blocked by incubating the sections with Carbo-Free™ Blocking Solution (Vector Laboratories, CAT#SP-5040) for 30 min at room temperature. VVA (1:2000, Vector Laboratories, CAT#B-1235-2) was used to incubate the sections overnight at 4 °C, then streptavidin HRP (1:10000, Abcam, CAT# ab7403) for 1h at room temperature.

### Immunofluorescence assay

After fixing in 4% paraformaldehyde for 10 min, cells were treated with 0.2% Triton X-100 for 10 min at room temperature, followed by blocking with goat serum at 37 °C for 60 min. The cells were then immunostained with the appropriate antibody or lectin. The following primary antibodies or lectins were used: anti-*GALNT1* (1:200; Abnova, CAT#H00002589-M10), anti-β-catenin (1:200; Proteintech, CAT#51067), anti-GM130 (1:200; Proteintech, CAT#11308-1-AP-150), anti-PDI (1:200; Cell Signaling Technology, CAT# 3501T), and VVA (1:200; Vector Laboratories, CAT# B-1235-2). Alexa Fluor 546-conjugated donkey anti‐rabbit IgG (1:1000, Thermo Fisher, CAT#A11003), DyLight™ 488 streptavidin (1:200, BioLegend, CAT# 405218), and Alexa Fluor 488-conjugated donkey anti-mouse IgG (1:1000, Thermo Fisher, CAT#A11001) were used as secondary antibodies. DAPI nucleic acid stain (Abcam, CAT# ab104139) was used to counterstain the sections. An inverted microscope was used to capture the images.

### Immunoprecipitation assays

The cells were lysed with IP Lysis Buffer (Pierce, CAT#87788), followed by immunoprecipitation with Protein A/G Magnetic Beads (Biomake, CAT#B23202-5) conjugated with CD44 immunoprecipitation specific polyclonal antibody. Then, they were incubated overnight at 4 °C and displayed via western blotting with biotin-conjugated VVA, as described above. Similarly, total cell lysates were immunoprecipitated using streptavidin-agarose (Sigma, CAT#S1638-1ML) conjugated with VVA immunoprecipitation-specific lectin. The immunocomplexes were then analyzed by western blotting.

### Luciferase reporter assay

Super TOP flash plasmids (Beyotime, CAT#D2506) and Renilla TK-luciferase vector were co-transfected into cells by Lipofectamine 6000 (Beyotime, CAT# C0526). The luciferase assay was performed using the Dual Luciferase Reporter Gene Assay Kit (YEASEN, CAT#11402ES60*) according to the manufacturer's protocol. And the firefly luciferase activity was normalized to Renilla.

### Cell viability and colony formation assay

A total of 1000-1500 cells per well were seeded in 96-well plates. Next, cell counting kit-8 (CCK-8) was used to determine the viability of cells incubated at 37 °C for 2 h at 0, 24, 48, 72, and 96 h, in accordance with the manufacturer's protocol. For colony formation assays, six-well plates containing 500 cells per well were used for colony formation experiments, and the cells were cultivated for two weeks. The colonies were then fixed with 4% paraformaldehyde for 15 min and stained with crystal violet (Beyotime Biotechnology, CAT#C0121) for 20 min.

### Cell migration and invasion assays

Transwell plates with 8-micrometer holes (Corning) were used to conduct migration and invasion assays. Cells were seeded in the top compartment with 0.2 ml serum-free medium, whereas 0.8 ml culture media with 10% fetal bovine serum was injected into the bottom chamber for the migration assay. When the cells grew for 24 h in an incubator, crystal violet staining was performed for 20 min with 4% paraformaldehyde for 15 min. Cotton swabs were used to remove the remaining unmigrated cells from the top layer. The protocols for the invasion experiments were roughly the same as for the migration assays, except for a 10% Matrigel (BD Biosciences) precoated in the top compartment and a doubled number of seeded cells. A 10× microscope (Olympus CKX53) was used to view and capture migrating or invading cells. ImageJ (X64) was used to count cells in the images.

### Animal studies

The TOPBIOTECH Animal Model Center's Experimental Animal Ethics Committee authorized *in vivo* studies, which were carried out in accordance with the institution's ethical rules for animal experimentation. Four-week-old female BALB/c mices were inoculated subcutaneously in the right anterior axilla with control (n = 6) or *GALNT1*-knockdown (n = 6) stably transfected SGC7901 cells (5.0 × 10^6^ cells). After 28 days, the mice were sacrificed. In the same time, four-week-old female BALB/c mice were injected with control (n = 7) or *GALNT1*-knockdown (n = 7) stably transfected SGC7901 cells (1.5 × 10^6^ cells) via the tail vein. After 7 weeks, the mice were euthanized, and the number of metastatic lung nodules was recorded.

### Statistical analysis

Unless otherwise stated, the data are presented as mean ± SD. Statistical Package for the Social Sciences (SPSS, version 26.0) and GraphPad Prism (Version 8.0) were used for all statistical analyses. To compare the two groups, the student's t-test was used. Paired t-tests were used to analyze paired GC tissues. The student's t-test was also used to examine the correlation between *GALNT1* or VVA expression and the clinicopathological characteristics of patients with GC. OS and DFS were examined by performing Kaplan-Meier curves, log-rank tests, and univariate/multivariate Cox regression analyses. Statistical significance was set at p< 0.05 (***p< 0.001, **p< 0.01, *p< 0.05).

## Results

### *GALNT1* is identified as a candidate target in GC by bioinformatics analysis

**Figure 1:**
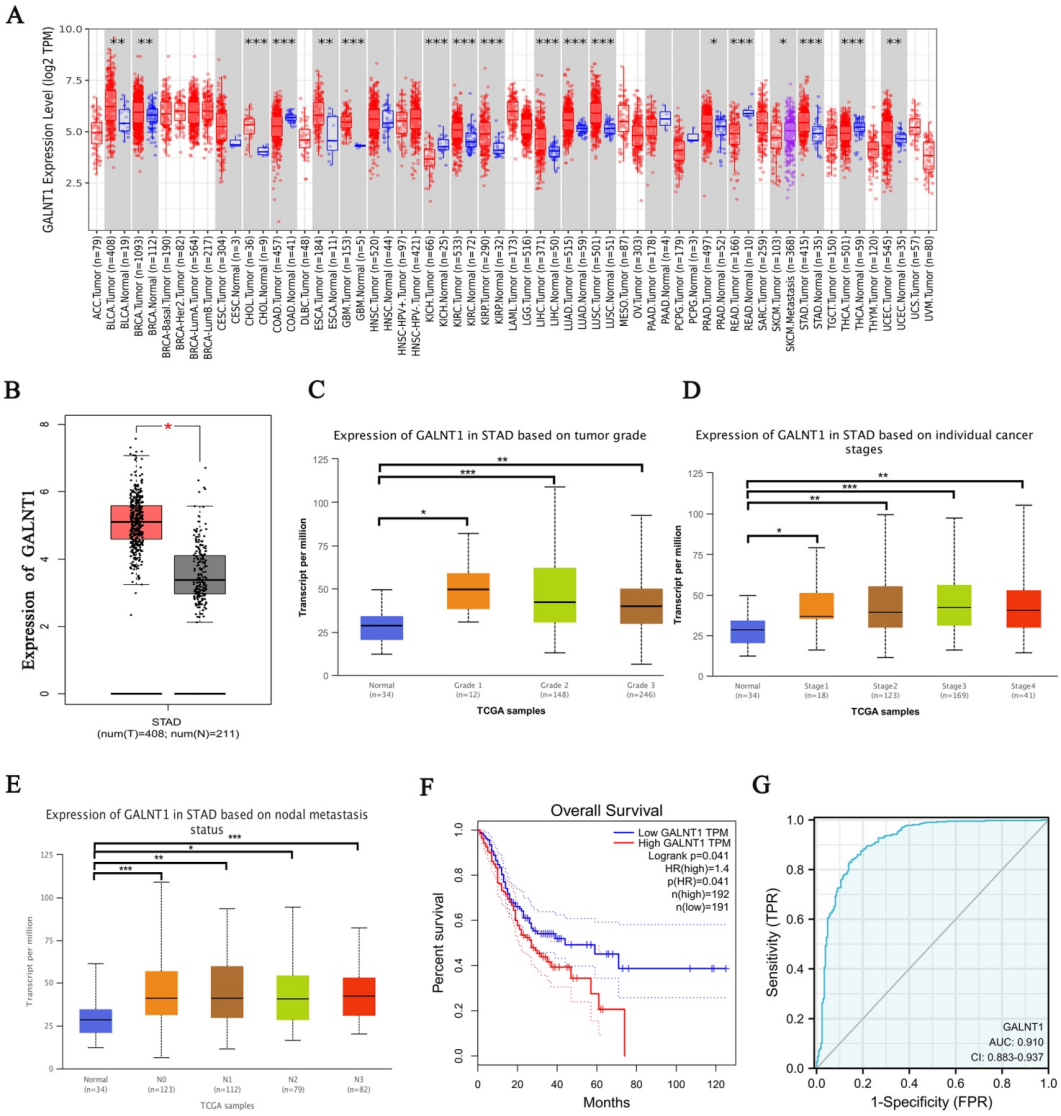
***GALNT1* as a candidate target in gastric cancer by bioinformatics analysis. A**, *GALNT1* expression in different types of human cancers. **B**, Analysis of *GALNT1* expression in gastric cancer using the GEPIA database. **C**-**E**, Analysis of *GALNT1* expression grouped by gastric cancer grade (**C**), pathological stages (**D**), and lymph node metastasis status (**E**). *** p<0.001. **F**, Overall survival comparing low and high expression of *GALNT1* in gastric cancer from TCGA datasets is shown. **G**, The receiver-operating characteristic (ROC) curve analysis of *GALNT1* in patients with gastric cancer from TCGA datasets.

To investigate the potential targets of O-glycosylation transferases in GC, we first investigated the expression of* GALNT1-20* in GC from The Cancer Genome Atlas (TCGA) database. Our study comprised 211 healthy tissues and 408 samples from patients with GC and their clinical medical records. Interestingly, the analysis revealed different expression patterns of these genes in the GC tissues. *GALNT1*, GALNT3, GALNT4, GALNT5, and GALNT6 were significantly higher in GC tissues, whereas GALNT10 expression was higher in normal tissues (Fig. S1, p<0.05). In the TCGA pan-cancer database, increased *GALNT1* expression was frequently observed in various cancers (Fig. 1A-B). Among these, we mainly focused on *GALNT1* because its function in GC remains elusive. Therefore, the emphasis of this study was on the impact and molecular mechanisms of *GALNT1* in GC.

Analysis of TCGA database by applying UALCAN, we examined *GALNT1* expression in relation to several clinicopathological parameters in patients with GC, including tumor grade (grade 1, 2, 3), tumor stage (stages I, II, III, and IV), and lymph node stage (N0 1, 2, and 3). The data indicated that *GALNT1* expression in any grade and stage was higher than that in the normal tissues (Fig. 1C-D). Moreover, *GALNT1* expression was related to lymph node metastasis, with the highest expression observed in N3 GC (Fig. 1E). Furthermore, the study suggested that patients with GC who had higher *GALNT1* expression had worse overall survival (OS) by using the Gene Expression Profiling Interactive Analysis (GEPIA) database (Fig. 1F). Interestingly, the receiver-operating characteristic (ROC) curve analysis of *GALNT1* in patients with GC from TCGA datasets confirmed that *GALNT1* has good diagnostic accuracy with an area under the curve (AUC) of 0.91 (95% CI: 0.883-0.937) (Fig. 1G). The above data indicate that *GALNT1* may be a candidate target in GC.

### Upregulated *GALNT1* expression promotes abnormal O-glycosylation in GC and correlates with aggressive clinicopathological features and poor survival

**Figure 2:**
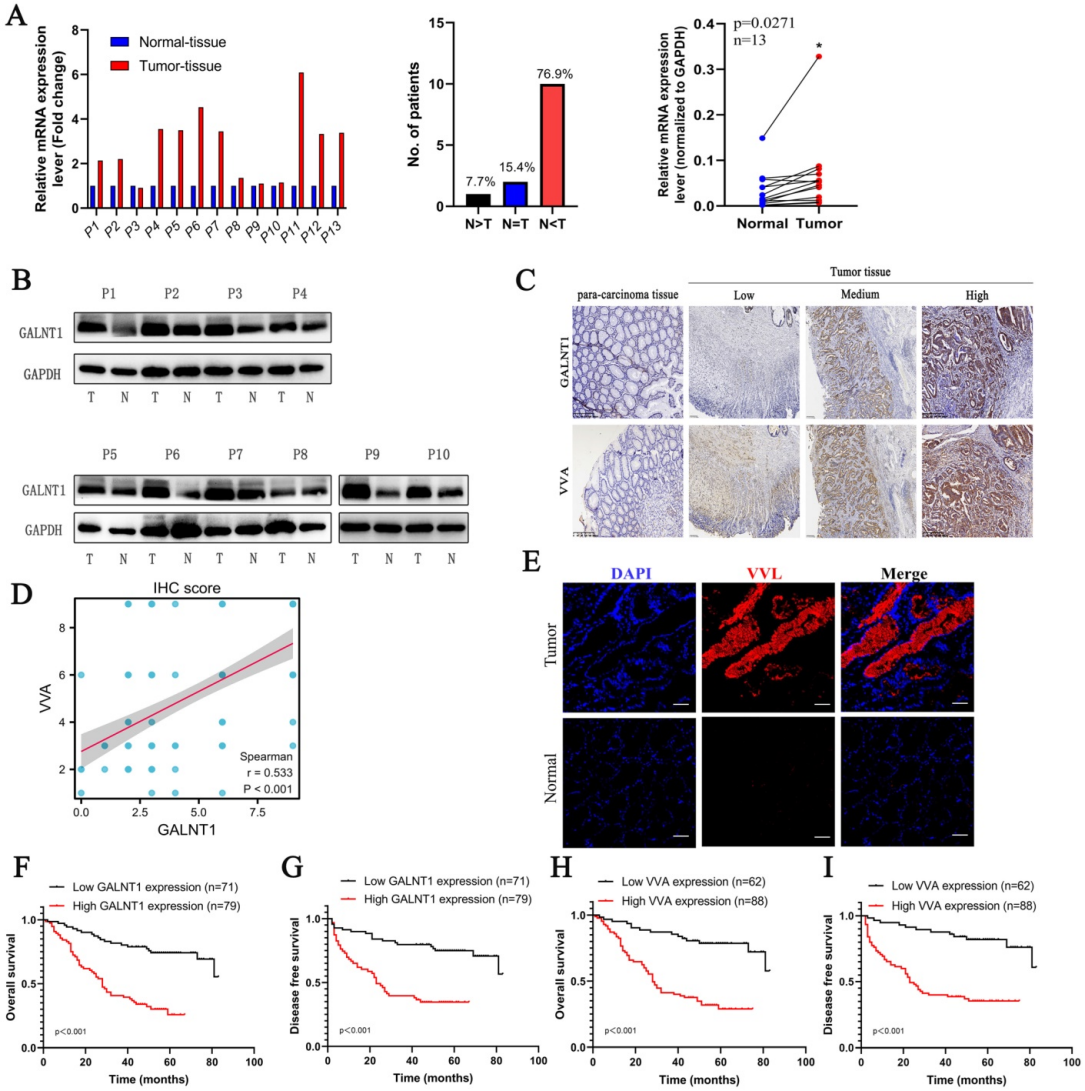
** The expression of GALNT1 and VVA in patients with gastric cancer. A**, The mRNA levels of *GALNT1* in 13 paired gastric cancer tissues and adjacent normal tissues (normalized to *GAPDH*). **B**, Western blot analysis depicting the GALNT1 levels in paired primary tumors and adjacent normal tissues. **C**, Representative immunohistochemical images of GALNT1 and VVA expression in samples and para-carcinoma tissues of patients with gastric cancer. **D**, Correlation analysis between GALNT1 and VVA immunohistochemistry score. **E**, Upregulated VVA expression in paired primary tumor tissues compared to adjacent normal tissues as examined by immunofluorescence assay. **F**-**I**, Kaplan-Meier analysis of overall survival and disease-free survival for patients with gastric cancer according to the immunohistochemistry of GALNT1 and VVA.

To confirm the above findings, paired GC tissues from 13 patients were analyzed using qRT-PCR. The results showed that 76.9% of GC tissues showed a positive increase in *GALNT1* mRNA expression compared to paired normal tissues (Fig. 2A). Consistently, western blotting analysis found that protein levels of *GALNT1* were elevated in GC tissues of matched specimens (Fig. 2B). To further investigate the relationship between *GALNT1* levels and clinicopathological characteristics, 150 GC tissue samples were used for immunohistochemical analyses (Fig. 2C). Immunohistochemical analysis of VVA in 150 GC specimens was performed to further investigate glycosylation levels (Fig. 2C). The results revealed that *GALNT1* and VVA were mainly expressed in the cytoplasm of GC cells and were considerably greater in primary tumor tissues than in neighboring normal tissues. Interestingly, correlation analyses between *GALNT1* and VVA levels based on the immunohistochemical scores showed that increased *GALNT1* levels promote abnormal O-glycosylation (Fig. 2D). Similarly, immunofluorescence analyses also showed that Tn levels were upregulated in GC cells (Fig. 2E).

In addition, we analyzed the association between *GALNT1* levels and clinicopathological factors (Table 1) and found that patients with larger tumor size (≥5 cm; p=0.002), Borrmann type (III/IV; p=0.005), pathological tumor-node-metastasis (pTNM) stage (III/IV; p<0.001), depth of invasion (T3/4; p=0.001), lymph node metastasis (p<0.001), distant metastasis (p=0.005), and lymphovascular invasion (LVI; p=0.022) tended to have higher levels of *GALNT1* in primary tumors (Table 1). Similar results were observed for VVA expression in GC (Table 1). To further understand the clinical importance of *GALNT1* and VVA in GC, we assessed the prognoses associated with *GALNT1* and VVA expression by Kaplan-Meier analysis. Patients with higher *GALNT1* and VVA expression had poorer OS and disease-free survival (DFS) than those with lower *GALNT1* and VVA expression levels (log-rank test, p<0.001; Fig. 2F-I). Multivariate Cox regression analysis indicated that tumor size (hazard ratio (HR): 2.098, 95%CI: 1.201-3.664, p=0.009; DFS: HR:2.057, 95%CI: 1.155-3.664, p=0.014), lymph node metastasis (OS: HR:2.965, 95%CI: 1.183-7.436, p=0.02; DFS: HR: 2.817, 95%CI: 1.116-7.114, p=0.028), and high *GALNT1* expression (OS: HR:2.458, 95%CI: 1.297-4.660, p=0.006; DFS: HR: 2.542, 95%CI: 1.335-4.841, p=0.005) were independent poor prognostic factors for OS and DFS (Tables 2 and 3). Collectively, these data suggest that *GALNT1* is frequently upregulated in GC and promotes abnormal O-glycosylation, which is associated with poor survival. This indicates that *GALNT1* is a potential prognostic marker for GC.

### *GALNT1* regulates proliferation, migration, and invasion of GC cells *in vitro*

**Figure 3:**
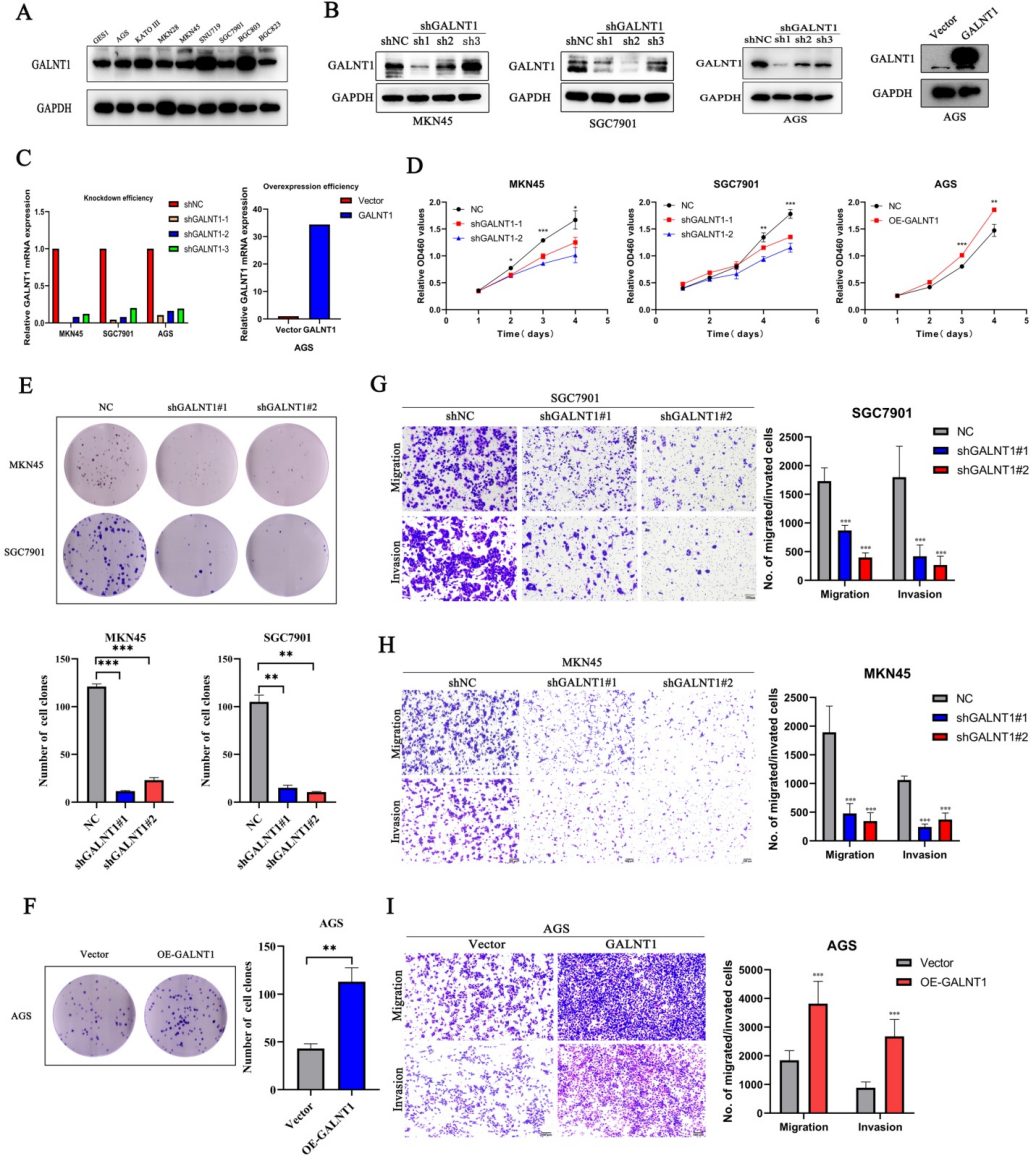
** GALNT1 promotes the growth and metastatic ability of gastric cancer cells *in vitro*. A,** GALNT1 expression in gastric cancer cell lines as examined by western blot analysis. **B**-**C,** Western blot (**B**) and qPCR (**C**) analysis depicting the silencing efficiency of *GALNT1* in MKN45, AGS, and SGC7901 cells as well as the overexpression of *GALNT1* in AGS cells. **D**-**F**, Cells growth ability after *GALNT1* knockdown and overexpression were determined by CCK8 assay (**D**) and colony formation assay (**E, F**). **G**-**I**, Knockdown of *GALNT1* decreased the abilities of migration and invasion of MKN45 and SGC7901 cells (**G, H**). Overexpression of *GALNT1* enhanced the abilities of migration and invasion of AGS cells (**I**). Data are shown as means ± S.D. *p<0.05, **p<0.01, ***p<0.001.

To better understand the effect of *GALNT1* in GC, we investigated phenotypic changes in GC cells after *GALNT1* knockdown or overexpression. We initially assessed the *GALNT1* levels in several cell lines using western blot assays (Fig. 3A). Then, *GALNT1* was stably knocked down in MKN45, SGC7901, and AGS cells, and overexpressed in AGS cells. The effectiveness of *GALNT1* knockdown and overexpression was examined by western blotting and qRT-PCR assays (Fig. 3B-C). Cell counting kit-8 (CCK8) assays revealed that *GALNT1* knockdown significantly impaired the growth of MKN45, SGC7901, and AGS cells, whereas *GALNT1* overexpression in AGS cells significantly increased cell proliferation (Fig. 3D, Fig. S2A). Similarly, colony formation assays further indicated that the clonogenic capacity of GC cells was suppressed after *GALNT1* deficiency, whereas overexpression of *GALNT1* in AGS cells yielded the opposite result (Fig. 3E-F, Fig. S2B). In addition, cell migration and invasion assays demonstrated that *GALNT1* knockdown significantly decreased the migration and invasion abilities of GC cells (Fig. 3G-H, Fig. S2C). Furthermore, *GALNT1* overexpression facilitated migration and invasion of AGS cells (Fig. 3I). Overall, these results suggest that *GALNT1* plays a crucial role in the proliferation, migration, and invasion of GC cells.

### *GALNT1* knockdown inhibits growth and metastasis of GC cells *in vivo*

**Figure 4:**
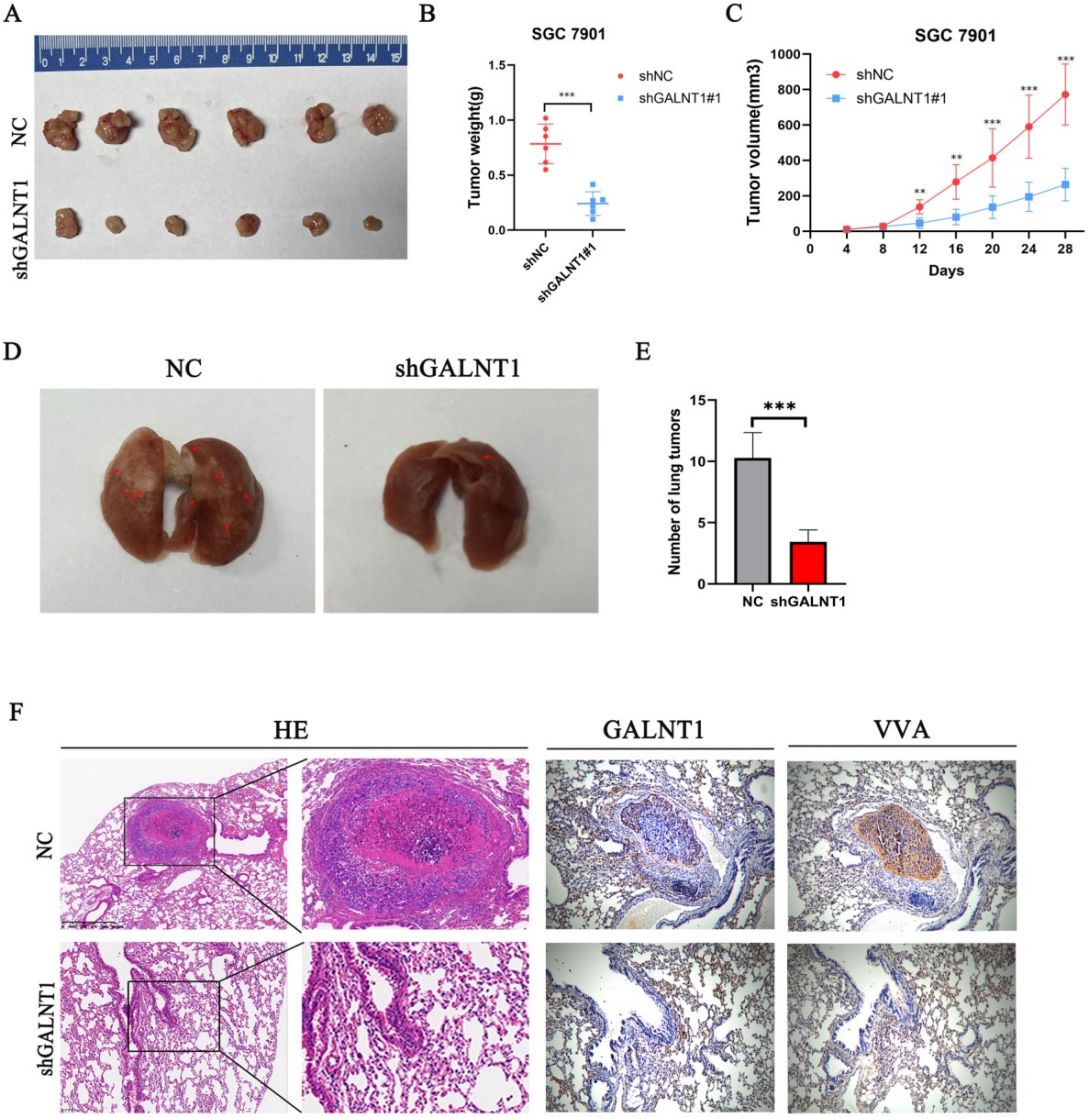
***GALNT1* regulates subcutaneous tumor formation and metastasis of gastric cancer in mice. A**, GALNT1 knockdown inhibited subcutaneous tumor formation in a nude mouse model. **B-C**, Tumor weight (**B**) and volume (**C**) analysis of subcutaneous tumors in nude mouse models. **D**, SGC7901 cells with control or *GALNT1* knockdown were injected into the tail veins of nude mice (n = 7 for each group). Representative images of lung taken seven weeks after injection. **E**, Quantification of metastatic lung tumors by hematoxylin & eosin. **F**, Representative images of metastatic lung tumors performed by hematoxylin & eosin staining and immunohistochemistry in mice. Data are shown as means ± S.D. *p<0.05, **p<0.01, ***p<0.001.

Subcutaneous tumor *in vivo* experiment was performed to investigate the function of *GALNT1* in driving tumorigenesis. Compared to the NC group, the *shGALNT1* group had a much smaller mean tumor volume and tumor weight (Fig. 4A-C), manifesting that targeted knockdown of *GALNT1* expression effectively inhibited tumor proliferation *in vivo*.

Metastasis is an important risk factor for a poor prognosis. As mentioned above, upregulated *GALNT1* is associated with lymph node metastasis and distant metastasis, and *GALNT1* can enhance the migration and invasion of GC cells *in vitro*. To further evaluate the metastatic ability of GC cells *in vivo*, we directly injected *GALNT1*-deficient SGC7901 cells and control cells into the tail veins of mice to examine the lung metastasis capacity of GC cells. Representative images of the lungs taken seven weeks after injection are shown in Fig. 4D. Mice implanted with *GALNT1*-deficient SGC7901 cells had fewer lung metastatic nodules than those in the control group, as shown by hematoxylin and eosin (H&E) staining (Fig. 4E). Furthermore, representative images showed that the expression levels of *GALNT1* and VVA were upregulated in the metastatic lung nodules following immunohistochemistry (Fig. 4F). These results indicate the vital role of *GALNT1* in enhancing the invasion and metastasis of GC.

### *GALNT1* activates the Wnt/β-catenin signaling in GC cells

**Figure 5:**
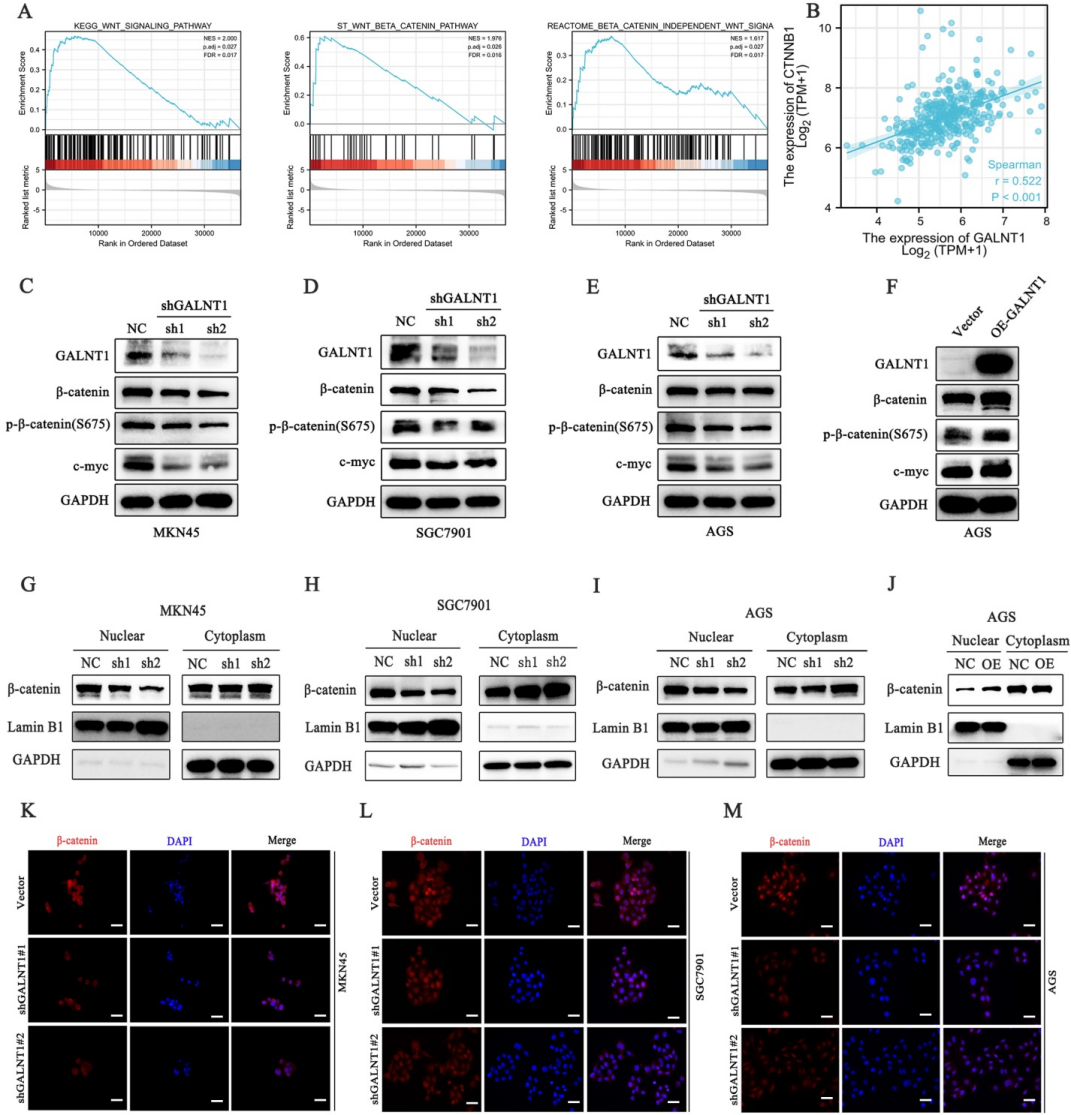
*GALNT1* positively regulates the β-catenin signaling pathway in gastric cancer cells. **A**, GSEA plots showing the Wnt/β-catenin pathways of differentially expressed genes altered by *GALNT1* in patients with gastric cancer from the TCGA database. **B**, Correlation of *GALNT1* and *β-catenin* in gastric cancer from TCGA database were analyzed. **C-F**, Western blotting analysis-based detection of protein expression of β-catenin, phospho-β-catenin (S675) and its downstream target c-Myc in MKN45 (**C**), SGC7901 (**D**) and AGS (**E**) cells after knockdown of *GALNT1*, as well as in the AGS cells after overexpression of *GALNT1* (**F**). **G-J**, Protein expression of β-catenin in the cytoplasm and nucleus of MKN45 (**G**), SGC7901 (**H**), and AGS (**I**) cells after knockdown of *GALNT1* as determined by western blot analysis, as well as AGS cells after overexpression of *GALNT1* (**J**). **K-M**, Nuclear translocation of β-catenin examined by immunofluorescence assays in *GALNT1*-silenced MKN45 (**K**), SGC7901 (**L**), and AGS (**M**) cells. Scale bar: 50μm.

To investigate the underlying mechanisms of *GALNT1* in GC development, we first performed gene set enrichment analysis (GSEA) analysis of TCGA data, which indicated that *GALNT1* was associated with the Wnt/β-catenin signaling pathway in GC (Fig. 5A). The aberrant Wnt/β-catenin signaling pathway is tightly linked to cell proliferation, invasion, and metastasis in multiple malignancies 21. To confirm the linkage between *GALNT1* and Wnt/β-catenin signaling, correlation analysis was conducted on TCGA data and revealed that *GALNT1* expression was positively associated with β-catenin expression in GC (r=0.522, p<0.001; Fig. 5B). Therefore, to further confirm whether Wnt/β-catenin is engaged in *GALNT1*-mediated pathways, the levels of key molecules in the pathway were examined by western blotting. We first silenced *GALNT1* expression using shRNA in multiple GC cell lines, including MKN45, SGC7901, and AGS, and discovered that the Wnt/β-catenin signaling-related molecules β-catenin and phospho-β-catenin (S675) had considerably lower levels, as did c-Myc, a downstream target (Fig. 5C-E). In contrast, *GALNT1* overexpression significantly increased the levels of these proteins (Fig. 5F). To investigate this further, nuclear/cytoplasmic separation was performed to detect β-catenin levels in the cytoplasm and nucleus. The expression levels of β-catenin in the nucleus were downregulated in *GALNT1*-knockdown cell lines of GC (Fig. 5G-I), whereas overexpression of *GALNT1* increased β-catenin nuclear expression (Fig. 5J). Moreover, immunofluorescence assays indicated that *GALNT1* deficiency reduced, whereas *GALNT1* overexpression upregulated the expression and nuclear signals of β-catenin (Fig. 5K-M). The luciferase reporter assay indicated that GALNT1 overexpression significantly increased the activity of β-catenin-driven transcription and vice versa (Fig. S3A-B). Collectively, these findings suggest that the Wnt/β-catenin signaling pathway is regulated by *GALNT1* overexpression.

### *GALNT1* mediates β-catenin signaling through O-glycosylation of CD44

**Figure 6:**
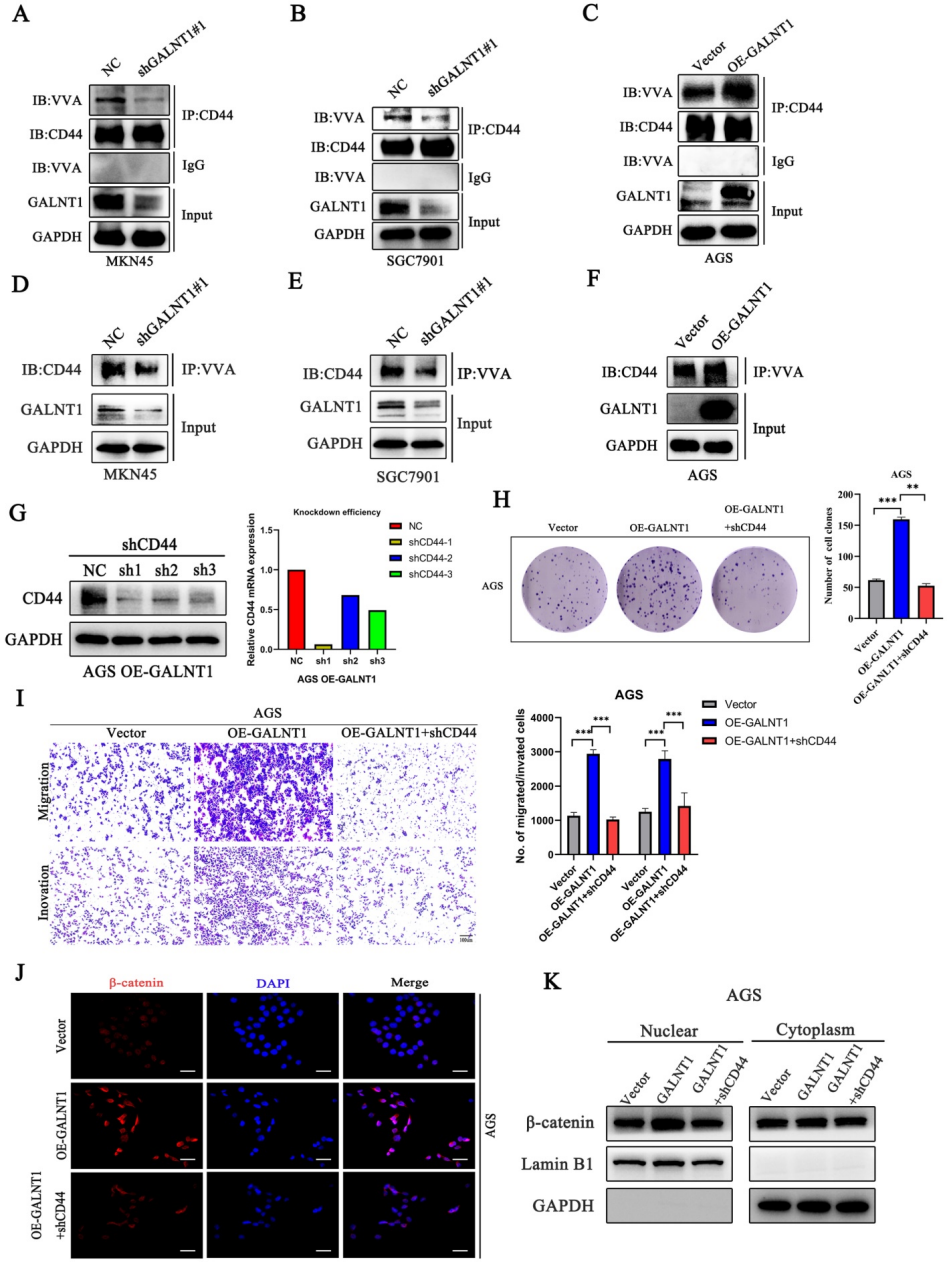
** GALNT1 positively regulates Wnt/β-catenin signaling pathway through O-glycosylation of CD44. A-C**, GALNT1 modification of O-glycosylation of CD44 measured by co-immunoprecipitation in gastric cancer cells. Cell lysates were immunoprecipitated (IP) for CD44, and then western blotted with biotin-conjugated VVA to distinguish Tn antigen expression. **D-F,** Lectin pull down glycoproteins using biotinylated VVA, and streptavidin agarose immunoblotted with anti-CD44 antibody. **G**, Western blot and qPCR analysis for the silencing efficiency of CD44 in AGS cells. **H**, The growth abilities of AGS cells after *GALNT1* overexpression and *CD44* knockdown were determined by colony formation assays. **I**, The migration and invasion abilities of AGS cells transfected with *OE-GALNT1* and *shCD44* measured by transwell assays. Representative images of the migrated or invaded cells and statistical graphs are shown. **J-K**, Immunofluorescence assay (**J**), and western blot analysis (**K**) depicting the nuclear translocation of β-catenin in AGS cells transfected with *OE-GALNT1* and *shCD44*. Scale bar: 50 µm. Data are shown as means ± S.D. *P<0.05, **P<0.01, ***P<0.001.

As *GALNT1* is an O-glycosyltransferase, O-glycosylation of proteins may be modified to mediate the Wnt/β-catenin signaling pathway in GC cells. A previous study has revealed that CD44 is a specific substrate for *GALNT1* 22. Therefore, we used NetOGlyc-4.0 to predict multiple O-glycosylation sites in CD44. Furthermore, we investigated the primary role of *GALNT1* in CD44 modified O-glycans in GC cells using VVA lectins, which recognize tumor-associated Tn antigens. To confirm whether *GALNT1* can modify O-glycosylation on CD44, cell lysates were immunoprecipitated (IP) for CD44 in GC cells with *GALNT1* silencing or overexpression, and western blotting with biotin-conjugated VVA was used to differentiate the levels of Tn antigen. These results show that *GALNT1* silencing reduced VVA binding to CD44 in MKN45, SGC7901, and AGS cells (Fig. 6A-B; Fig. S4A). Conversely, *GALNT1* overexpression increased the binding of VVA to CD44 in AGS cells (Fig. 6C). To further verify these results, lectin pull-down of glycoproteins using biotinylated VVA and streptavidin agarose were immunoblotted (IB) with an anti-CD44 antibody. Consistently, western blot analysis indicated that *GALNT1* silencing downregulated (Fig. 6D-E; Fig. S4B), whereas overexpression of *GALNT1* enhanced the expression of CD44 in glycoproteins pulled down by VVA lectin (Fig. 6F). This finding suggests that *GALNT1* plays a crucial role in modifying O-glycans on CD44 in GC cells.

To investigate the mechanism by which *GALNT1* regulates the Wnt/β-catenin signaling pathway through O-glycosylation of CD44, we conducted a rescue assay in AGS cells overexpressing *GALNT1* and the corresponding control cells. We first stably knocked down CD44 in AGS cells overexpressing *GALNT1*, and then western blot and qPCR analyses were utilized to investigate the efficiency of knockdown (Fig. 6G). As shown in Fig. 6H, clonogenic capacity was reversed by CD44 knockdown in AGS cells overexpressing *GALNT1*. Consistently, CD44 silencing in AGS cells with *GALNT1* overexpression reversed the favorable effects of *GALNT1* on migration and invasion (Fig. 6I). Moreover, immunofluorescence assays revealed that CD44 knockdown abolished the favorable effects of *GALNT1* on the nuclear signals of β-catenin in AGS cells (Fig. 6J). Meanwhile, nuclear/cytoplasmic separation analysis also revealed that nuclear β-catenin signals were reversed by CD44 knockdown in AGS cells overexpressing *GALNT1* (Fig. 6K). Collectively, these findings suggest that *GALNT1* mediates the β-catenin signaling pathway through the O-glycosylation of CD44.

### *GALNT1*/Tn antigen is relocated into the ER

**Figure 7:**
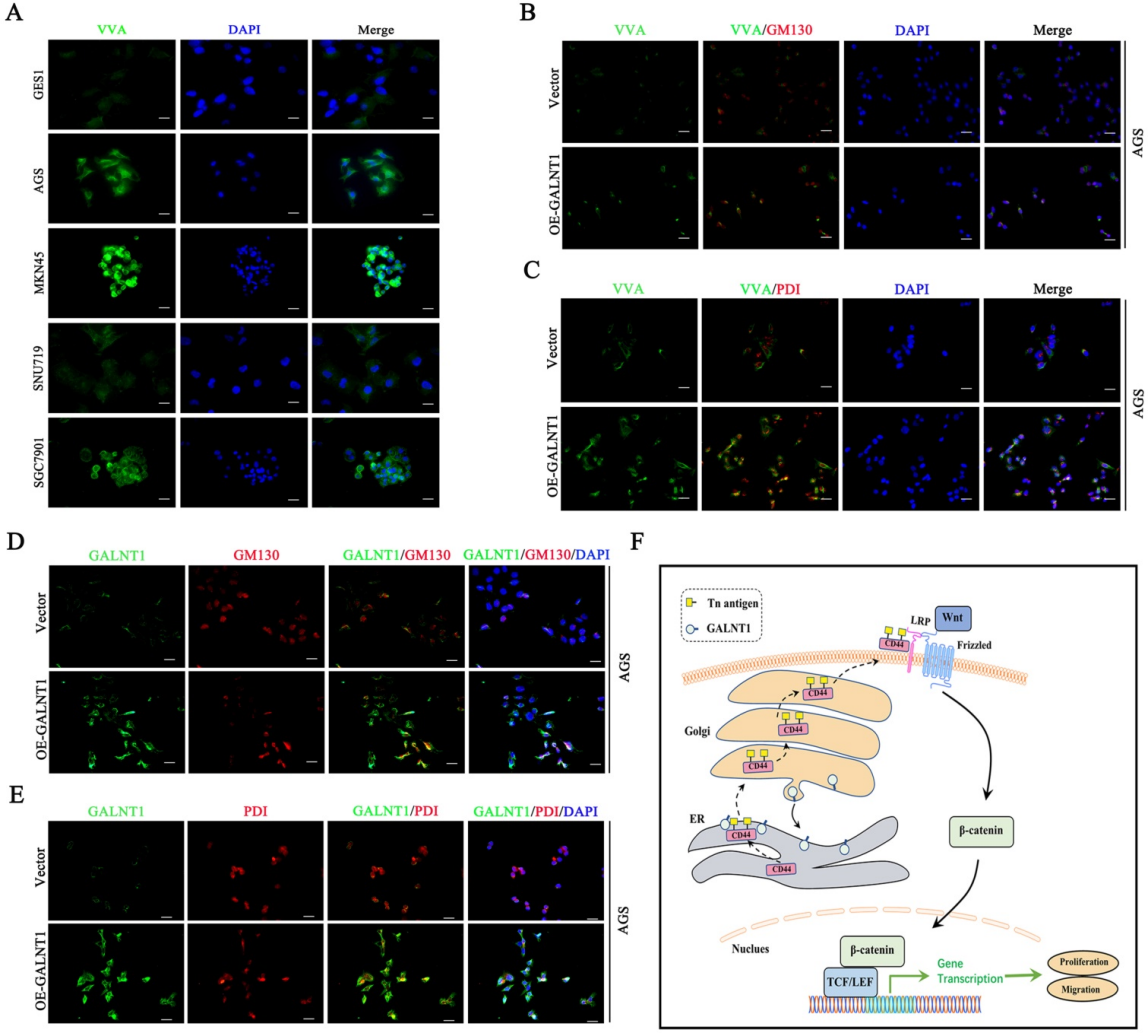
** GALNT1/Tn translocates to the ER. A**, Immunofluorescence of Tn antigen in multiple gastric cancer cells. **B**, Co-staining Tn antigen (VVA, green) and GM130 (red) in the indicated cells. **C**, Co-staining Tn antigen (VVA, green) and PDI (red) in the indicated cells. **D**, Colocalization of GALNT1 (green) and GM130 (red) in AGS cells after overexpression of *GALNT1*. **E**, Colocalization of GALNT1 (green) and PDI (red) in AGS cells after overexpression of *GALNT1*. Scale bar: 50μm. **F**, Schematic illustration of the working hypothesis.

Interestingly, immunofluorescence analysis of multiple GC cells showed that Tn antigen expression was upregulated in the aggressive GC cells MKN45 and SGC7901, whereas it was decreased in less-invasive AGS and SNU719 cells (Fig. 7A). This was similar to our western blot findings of *GALNT1* expression levels in GC cells. Tn antigen expression is associated with the initiation of O-glycosylation, and the Golgi apparatus is the primary site of O-glycosylation 16. Recent studies have found that O-glycosylation initiated by *GALNT1* is relocated from the Golgi apparatus to the endoplasmic reticulum (ER) during tumor progression and metastasis (GALA pathway) 8, 17, 18. Therefore, to verify the mechanism, we first investigated the interaction between the Tn antigen (VVA, green) and Golgi-marker GM130 (red) or ER-marker PDI in AGS cells overexpressing *GALNT1*. Strikingly, we found that abundant Tn antigen was located in the ER (Fig. 7B-C). In addition, immunofluorescence analysis of *GALNT1* with GM130 or PDI also indicated that *GALNT1* was located in the Golgi as well as obviously ER in AGS cells overexpressing *GALNT1* (Fig. 7D-E). Collectively, the initiation of O-glycosylation is associated with the relocation of *GALNT1* to the ER during tumor progression and metastasis.

## Discussion

Previous studies have indicated that *GALNT1* is upregulated in multiple malignancies, including hepatocellular carcinoma, breast cancer, and bladder cancer, and is strongly associated with tumor progression 17, 18, 23. However, the function and detailed molecular mechanism of *GALNT1* in GC remain largely unknown. In the present study, we confirmed that *GALNT1* expression is commonly upregulated in GC and that it is closely associated with the pTNM stage, depth of invasion, metastasis, and poor survival. Moreover, we revealed its involvement in maintaining malignancy and facilitation of metastasis. Mechanistic investigations suggest that overexpression of *GALNT1* in GC cells modifies the O-glycosylation of CD44, stimulating the Wnt/β-catenin signaling pathway to promote the progression and metastasis of GC. The main conclusions of this study are shown in Fig. 7F. This is the first study to determine that *GALNT1* plays a crucial role in GC progression.

Although significant advances have been made in detecting and treating GC, metastasis remains the primary reason for the high mortality of patients with GC 2. Hence, it is essential to understand the detailed molecular mechanisms of metastasis and develop more effective and targeted reagents for the treatment of GC. Bioinformatics analysis showed that *GALNT1* is overexpressed in GC as well as other malignant tumors, including breast cancer, bladder cancer, and hepatocellular carcinoma. Our findings were consistent with the previously reported data 17, 18, 23. These results suggest that *GALNT1* may be a crucial oncogene during tumor evolution. Moreover, we further confirmed that *GALNT1* is frequently upregulated in GC and promotes abnormal O-glycosylation, which is associated with poor survival, by analyzing the clinicopathological features of GC patients. Hence, to further confirm the effects of *GALNT1* on gastric carcinogenesis and progression, the effects of upregulating and downregulating *GALNT1* on cell function were examined. We further demonstrated that *GALNT1* expression affects the malignant and metastatic phenotypes of GC cells *in vivo* and *in vitro*. These findings suggest that *GALNT1* may be a valuable biomarker and candidate target for new GC treatments.

In numerous cancers, the Wnt/β-catenin signaling pathway is often inappropriately activated 24, 25. This pathway plays a vital role in embryonic development, and regulates the development, regulation, and survival of cancer cells 26, 27. In GC, the Wnt/β-catenin signaling pathway contributes to cancer development and is implicated in recurrence and poor survival 28-30. Consistently, in the present study, we found that *GALNT1* knockdown reduced the protein expression of β-catenin, p-β-catenin (S675), and its downstream target c-Myc in GC cells, whereas *GALNT1* overexpression increased the expression of these proteins. Moreover, we revealed that silencing *GALNT1* inhibited, whereas *GALNT1* overexpression promoted β-catenin translocation into the nucleus in GC cells. Collectively, these results indicate that *GALNT1* positively regulates the Wnt/β-catenin signaling pathway in GC cells. Therefore, understanding the detailed regulatory mechanisms involved is essential.

*GALNT1* plays a crucial role in the biosynthesis of O-glycans such as the Tn antigen, which is identified by VVA lectin. In breast and liver cancer, previous studies have indicated that higher Tn antigen levels were observed in cancerous tissues than in healthy tissues and were positively linked to progressive tumors and poor survival 17, 18. These results are consistent with our finding that Tn antigen levels are increased in GC tissues and confer a poorer prognosis. Cancer vaccines based on short O-glycans have been developed as novel cancer therapies 31. Glycoproteins modified with short O-glycans frequently affect the function of cancer cells and their antigenic properties, thereby promoting the development of invasion and metastasis capabilities 32. Moreover, new discoveries have revealed that GALNTs relocate from the Golgi apparatus to the ER to initiate O-glycosylation 8, 17, 18, 33. We also observed this translocation of *GALNT1* to the ER. However, the role of short O-glycans in GC progression remains unclear. Nonetheless, our study revealed that *GALNT1* is the main GalNAc transferase in GC and that it promotes abnormal O-glycosylation.

In the present study, we found that *GALNT1* could modify CD44 O-glycosylation in GC cells. Consistently, a previous study revealed that CD44 is a specific substrate for *GALNT1* 22. Moreover, CD44 has multiple O-glycosylation sites, as predicted by NetOGlyc-4.0. CD44 transmembrane glycoproteins influence cell growth, survival, differentiation, and motility, and are correlated with malignant tumors 34, 35. Many studies have reported that CD44 is aberrantly expressed in several human tumors, and it plays a crucial role in cancer metastasis and tumor growth 35-40. In GC, CD44 has also been identified as an oncogene with high malignant potential, which can promote metastasis 41, 42. In addition, recent research has shown that CD44 acts as a positive regulator of the Wnt/β-catenin signaling pathway 43. Therefore, we conducted a rescue assay in AGS cells overexpressing *GALNT1* and the corresponding control cells to investigate the mechanisms by which the *GALNT1*-mediated O-glycosylation of CD44 regulates the Wnt/β-catenin signaling pathway. We found that CD44 silencing in AGS cells with *GALNT1* overexpression reversed the positive effects of *GALNT1* on the migration, invasion, and nuclear signals of β-catenin. To our knowledge, this is the first study to report that *GALNT1* regulates the Wnt/β-catenin signaling pathway through the O-glycosylation of CD44 in GC cells. Further validation of the specific O-glycosylation sites in CD44 would help understand the regulatory mechanism of the Wnt/β-catenin signaling pathway.

## Conclusion

*GALNT1* is upregulated in GC and promotes abnormal O-glycosylation of CD44, thereby activating the Wnt/β-catenin signaling pathway and regulating the malignant behavior of GC cells. This study provides insights into the pathophysiological role of *GALNT1* and the significance of abnormal O-glycosylation in GC tumor progression. These findings may pave the way for developing novel therapeutic agents for GC.

## Supplementary Material

Supplementary figures and tables.

## Figures and Tables

**Table 1:** Associations of *GALNT1* and VVA expression with clinical parameters in GC patients

Characteristic	*GALNT1*			VVA		
	Low (%)	High (%)	*P*	Low (%)	High (%)	*P*
**Age (years)**			0.403			0.471
<60	32(43.8)	41(56.2)		28(38.4)	45(61.6)	
≥60	39(50.6)	38(49.4)		34(44.2)	43(55.8)	
**Gender**			0.327			0.418
Male	46(50.5)	45(49.5)		40(44.0)	51(56.0)	
Female	25(42.4)	34(57.6)		22(37.3)	37(26.7)	
**Tumor size**			**<0.001**			**0.015**
≤5 cm	52(60.5)	34(39.5)		43(50.0)	43(50.0)	
>5 cm	19(30.2)	31(69.8)		19(30.2)	44(69.8)	
**Borrmann type**			**0.005**			**0.023**
I-II	20(76.9)	6(23.1)		15(57.7)	11(42.3)	
III-IV	40(45.4)	48(54.5)		29(33.0)	59(67.0)	
**Differentiation**			0.666			0.617
Well+ moderate	19(55.9)	15(44.1)		16(47.1)	18(52.9)	
poor	49(51.6)	46(48.4)		40(42.1)	55(57.9)	
**pTNM stage**			**<0.001**			**<0.001**
I	20(74.1)	7(25.9)		20(74.1)	7(25.9)	
II	28(87.5)	4(12.5)		21(65.6)	11(34.4)	
III	21(27.6)	55(72.4)		18(23.7)	58(76.3)	
IV	2(13.3)	13(86.7)		3(20.0)	12(80.0)	
**Depth of invasion**			**0.001**			**<0.001**
T1/2	24(72.7)	9(27.3)		24(72.7)	9(27.3)	
T3/4	47(40.2)	70(59.8)		38(32.5)	79(67.5)	
**Lymph node metastasis**			**<0.001**			**<0.001**
N0	41(82.0)	9(18.0)		35(70.0)	15(30.0)	
N+	30(30.0)	70(70.0)		27(27.0)	73(73.0)	
**Distant metastasis**			**0.005**			0.077
M0	69(51.1)	66(48.9)		59(43.7)	76(56.3)	
M1	2(13.3)	13(86.7)		3(20.0)	12(80.0)	
**CEA level (μg/L)**			0.319			0.912
≤5	61(49.2)	63(50.8)		51(41.1)	73(58.1)	
>5	10(38.5)	16(61.5)		11(42.3)	15(57.7)	
**LVI**			**0.011**			**0.009**
Yes	12(32.4)	25(67.6)		9(24.3)	28(75.7)	
No	57(57.0)	43(43.0)		49(24.3)	51(75.7)	
**PNI**			0.949			0.917
Yes	8(50.0)	8(50.0)		7(43.8)	9(56.3)	
No	60(50.8)	58(49.2)		50(42.4)	68(57.6)	

Bold indicates statistically significant value.

**Table 2:** Univariate and multivariate analyses for OS in GC patients

Variable	Univariate			Multivariate		
	HR	95%CI	*P*	HR	95%CI	*P*
**Age (years)**						
≥60 vs. <60	0.868	0.553-1.363	0.539			
**Gender**						
Male vs. Female	0.731	0.464-1.152	0.178			
**Tumor size**						
>5 cm vs. ≤5 cm	3.201	1.995-5.137	**<0.001**	2.098	1.201-3.664	**0.009**
**Borrmann type**						
III-IV vs. I-II	5.506	1.992-15.219	**0.001**	2.412	0.825-7.050	0.108
**Differentiation**						
Poor vs. Well+ moderate	1.717	0.908-3.246	0.096			
**Depth of invasion**						
T3-4 vs. T1-2	15.71	3.851-64.095	**<0.001**			
**Lymph node metastasis**						
N+ vs. N0	7.871	3.609-17.169	**<0.001**	2.965	1.183-7.436	**0.020**
**Distant metastasis**						
M1 vs. M0	3.011	1.644-5.514	**<0.001**			
**CEA level (μg/L)**						
>5 vs. ≤5	1.787	1.059-3.015	**0.03**			
**LVI**						
Present vs. none	2.980	1.816-4.888	**<0.001**			
**PNI**						
Present vs. none	1.388	0.684-2.817	0.364			
***GALNT1***						
High vs. Low	4.406	2.574-7.541	**<0.001**	2.458	1.297-4.660	**0.006**

Bold indicates statistically significant value.

**Table 3:** Univariate and multivariate analyses for DFS in GC patients

Variable	Univariate			Multivariate		
	HR	95%CI	*P*	HR	95%CI	*P*
**Age (years)**						
≥60 vs. <60	0.924	0.571-1.493	0.746			
**Gender**						
Male vs. Female	0.742	0.456-1.207	0.229			
**Tumor size**						
>5 cm vs. ≤5 cm	3.716	2.250-6.137	**<0.001**	2.057	1.155-3.664	**0.014**
**Borrmann type**						
III-IV vs. I-II	5.645	2.043-15.602	**0.001**	2.572	0.885-7.475	0.083
**Differentiation**						
Poor vs. Well+ moderate	1.731	0.916-3.272	0.091			
**Depth of invasion**						
T3-4 vs. T1-2	29.55	4.096-213.16	**0.001**			
**Lymph node metastasis**						
N+ vs. N0	7.604	3.466-16.683	**<0.001**	2.817	1.116-7.114	**0.028**
**Distant metastasis**						
M1 vs. M0	3.287	1.184-9.124	**0.022**			
**CEA level (μg/L)**						
>5 vs. ≤5	1.872	1.064-3.296	**0.030**			
**LVI**						
Present vs. none	3.217	1.923-5.380	**<0.001**	1.680	0.943-2.994	0.079
**PNI**						
Present vs. none	1.093	0.469-2.546	0.836			
***GALNT1***						
High vs. Low	4.259	2.434-7.454	**<0.001**	2.542	1.335-4.841	**0.005**

Bold indicates statistically significant value.

## Abbreviations

*GALNT1*: N-acetylgalactosamine-transferase 1
GC: gastric cancer
VVA: vicia villosa
GalNAc: N-acetylgalactosamine
Ser: serine
Thr: threonine
ER: endoplasmic reticulum
GALA: GALNTs activation
MUC1: mucin 1
OPN: osteopontin
MMP14: matrix metalloproteinase-14
TCGA: The Cancer Genome Atlas
ROC: receiver-operating characteristic
AUC: area under the curve
pTNM: pathological tumor-node-metastasis
LVI: lymphovascular invasion
OS: overall survival
GEPIA: Gene Expression Profiling Interactive Analysis
DFS: disease-free survival
PBS: phosphate-buffered saline
PVDF: polyvinylidene fluoride
NC: nitrocellulose
DAB: 3,3'-diaminobenzidine
H&E: hematoxylin and eosin
GSEA: gene set enrichment analysis
IB: immunoblot
sh*GALNT1*: *GALNT1* knockdown
shCD44: CD44 knockdown
qRT-PCR: quantitative real-time PCR
IHC: immunohistochemistry
HRP: horseradish peroxidase
CCK-8: cell counting kit-8

## References

[1] Sung H, Ferlay J, Siegel RL, Laversanne M, Soerjomataram I, Jemal A (2021). Global Cancer Statistics 2020: GLOBOCAN Estimates of Incidence and Mortality Worldwide for 36 Cancers in 185 Countries. CA Cancer J Clin.

[2] Luebeck EG, Curtius K, Jeon J, Hazelton WD (2013). Impact of tumor progression on cancer incidence curves. Cancer Res.

[3] Pinho SS, Reis CA (2015). Glycosylation in cancer: mechanisms and clinical implications. Nat Rev Cancer.

[4] Fuster MM, Esko JD (2005). The sweet and sour of cancer: glycans as novel therapeutic targets. Nat Rev Cancer.

[5] Bard F, Chia J (2016). Cracking the Glycome Encoder: Signaling, Trafficking, and Glycosylation. Trends Cell Biol.

[6] Ju T, Otto VI, Cummings RD (2011). The Tn antigen-structural simplicity and biological complexity. Angew Chem Int Ed Engl.

[7] Duarte HO, Freitas D, Gomes C, Gomes J, Magalhães A, Reis CA (2016). Mucin-Type O-Glycosylation in Gastric Carcinogenesis. Biomolecules.

[8] Gill DJ, Tham KM, Chia J, Wang SC, Steentoft C, Clausen H (2013). Initiation of GalNAc-type O-glycosylation in the endoplasmic reticulum promotes cancer cell invasiveness. Proc Natl Acad Sci U S A.

[9] Springer GF (1984). T and Tn, general carcinoma autoantigens. Science.

[10] Chia J, Tham KM, Gill DJ, Bard-Chapeau EA, Bard FA (2014). ERK8 is a negative regulator of O-GalNAc glycosylation and cell migration. Elife.

[11] Bennett EP, Mandel U, Clausen H, Gerken TA, Fritz TA, Tabak LA (2012). Control of mucin-type O-glycosylation: a classification of the polypeptide GalNAc-transferase gene family. Glycobiology.

[12] Liu Y, Liu H, Yang L, Wu Q, Liu W, Fu Q (2017). Loss of N-Acetylgalactosaminyltransferase-4 Orchestrates Oncogenic MicroRNA-9 in Hepatocellular Carcinoma. J Biol Chem.

[13] Taniuchi K, Cerny RL, Tanouchi A, Kohno K, Kotani N, Honke K (2011). Overexpression of GalNAc-transferase GalNAc-T3 promotes pancreatic cancer cell growth. Oncogene.

[14] Li Z, Yamada S, Inenaga S, Imamura T, Wu Y, Wang KY (2011). Polypeptide N-acetylgalactosaminyltransferase 6 expression in pancreatic cancer is an independent prognostic factor indicating better overall survival. Br J Cancer.

[15] Wu C, Guo X, Wang W, Wang Y, Shan Y, Zhang B (2010). N-Acetylgalactosaminyltransferase-14 as a potential biomarker for breast cancer by immunohistochemistry. BMC Cancer.

[16] Röttger S, White J, Wandall HH, Olivo JC, Stark A, Bennett EP (1998). Localization of three human polypeptide GalNAc-transferases in HeLa cells suggests initiation of O-linked glycosylation throughout the Golgi apparatus. J Cell Sci.

[17] Fang R, Xu F, Shi H, Wu Y, Cao C, Li H (2020). LAMTOR5 raises abnormal initiation of O-glycosylation in breast cancer metastasis via modulating *GALNT1* activity. Oncogene.

[18] Nguyen AT, Chia J, Ros M, Hui KM, Saltel F, Bard F (2017). Organelle Specific O-Glycosylation Drives MMP14 Activation, Tumor Growth, and Metastasis. Cancer Cell.

[19] Gerken TA, Raman J, Fritz TA, Jamison O (2006). Identification of common and unique peptide substrate preferences for the UDP-GalNAc:polypeptide alpha-N-acetylgalactosaminyltransferases T1 and T2 derived from oriented random peptide substrates. J Biol Chem.

[20] Li C, Yang Z, Du Y, Tang H, Chen J, Hu D (2014). BCMab1, a monoclonal antibody against aberrantly glycosylated integrin α3β1, has potent antitumor activity of bladder cancer *in vivo*. Clin Cancer Res.

[21] Lim SK, Lu SY, Kang SA, Tan HJ, Li Z, Adrian Wee ZN (2016). Wnt Signaling Promotes Breast Cancer by Blocking ITCH-Mediated Degradation of YAP/TAZ Transcriptional Coactivator WBP2. Cancer Res.

[22] Narimatsu Y, Joshi HJ, Schjoldager KT, Hintze J, Halim A, Steentoft C (2019). Exploring Regulation of Protein O-Glycosylation in Isogenic Human HEK293 Cells by Differential O-Glycoproteomics. Mol Cell Proteomics.

[23] Li C, Du Y, Yang Z, He L, Wang Y, Hao L (2016). *GALNT1*-Mediated Glycosylation and Activation of Sonic Hedgehog Signaling Maintains the Self-Renewal and Tumor-Initiating Capacity of Bladder Cancer Stem Cells. Cancer Res.

[24] Pan J, Fang S, Tian H, Zhou C, Zhao X, Tian H (2020). lncRNA JPX/miR-33a-5p/Twist1 axis regulates tumorigenesis and metastasis of lung cancer by activating Wnt/β-catenin signaling. Mol Cancer.

[25] Li Y, Chen F, Shen W, Li B, Xiang R, Qu L (2020). WDR74 induces nuclear β-catenin accumulation and activates Wnt-responsive genes to promote lung cancer growth and metastasis. Cancer Lett.

[26] Nusse R, Clevers H (2017). Wnt/β-Catenin Signaling, Disease, and Emerging Therapeutic Modalities. Cell.

[27] Clevers H, Nusse R (2012). Wnt/β-catenin signaling and disease. Cell.

[28] Ji L, Qian W, Gui L, Ji Z, Yin P, Lin GN (2020). Blockade of β-Catenin-Induced CCL28 Suppresses Gastric Cancer Progression via Inhibition of Treg Cell Infiltration. Cancer Res.

[29] Wang JB, Wang ZW, Li Y, Huang CQ, Zheng CH, Li P (2017). CDK5RAP3 acts as a tumor suppressor in gastric cancer through inhibition of β-catenin signaling. Cancer Lett.

[30] Ganesan K, Ivanova T, Wu Y, Rajasegaran V, Wu J, Lee MH (2008). Inhibition of gastric cancer invasion and metastasis by PLA2G2A, a novel beta-catenin/TCF target gene. Cancer Res.

[31] Astronomo RD, Burton DR (2010). Carbohydrate vaccines: developing sweet solutions to sticky situations?. Nat Rev Drug Discov.

[32] Brockhausen I (2006). Mucin-type O-glycans in human colon and breast cancer: glycodynamics and functions. EMBO Rep.

[33] Gill DJ, Clausen H, Bard F (2011). Location, location, location: new insights into O-GalNAc protein glycosylation. Trends Cell Biol.

[34] Zöller M (2011). CD44: can a cancer-initiating cell profit from an abundantly expressed molecule?. Nat Rev Cancer.

[35] Ponta H, Sherman L, Herrlich PA (2003). CD44: from adhesion molecules to signalling regulators. Nat Rev Mol Cell Biol.

[36] Jordan AR, Racine RR, Hennig MJ, Lokeshwar VB (2015). The Role of CD44 in Disease Pathophysiology and Targeted Treatment. Front Immunol.

[37] Kim H, Yang XL, Rosada C, Hamilton SR, August JT (1994). CD44 expression in colorectal adenomas is an early event occurring prior to K-ras and p53 gene mutation. Arch Biochem Biophys.

[38] Wielenga VJ, Heider KH, Offerhaus GJ, Adolf GR, van den Berg FM, Ponta H (1993). Expression of CD44 variant proteins in human colorectal cancer is related to tumor progression. Cancer Res.

[39] Szatanek R, Baj-Krzyworzeka M (2021). CD44 and Tumor-Derived Extracellular Vesicles (TEVs). Possible Gateway to Cancer Metastasis. Int J Mol Sci.

[40] Ouhtit A, Rizeq B, Saleh HA, Rahman MM, Zayed H (2018). Novel CD44-downstream signaling pathways mediating breast tumor invasion. Int J Biol Sci.

[41] Nguyen PH, Giraud J, Chambonnier L, Dubus P, Wittkop L, Belleannée G (2017). Characterization of Biomarkers of Tumorigenic and Chemoresistant Cancer Stem Cells in Human Gastric Carcinoma. Clin Cancer Res.

[42] Chen T, Yang K, Yu J, Meng W, Yuan D, Bi F (2012). Identification and expansion of cancer stem cells in tumor tissues and peripheral blood derived from gastric adenocarcinoma patients. Cell Res.

[43] Schmitt M, Metzger M, Gradl D, Davidson G, Orian-Rousseau V (2015). CD44 functions in Wnt signaling by regulating LRP6 localization and activation. Cell Death Differ.

