# Supplementary material for: GALNT1 Enhances Malignant Phenotype of Gastric Cancer via Modulating CD44 Glycosylation to Activate the Wnt/β-catenin Signaling Pathway
Source: Int J Biol Sci. 2022 Oct 17;18(16):6068–83. doi: 10.7150/ijbs.73431 (PMC9682532; doi:10.7150/ijbs.73431)

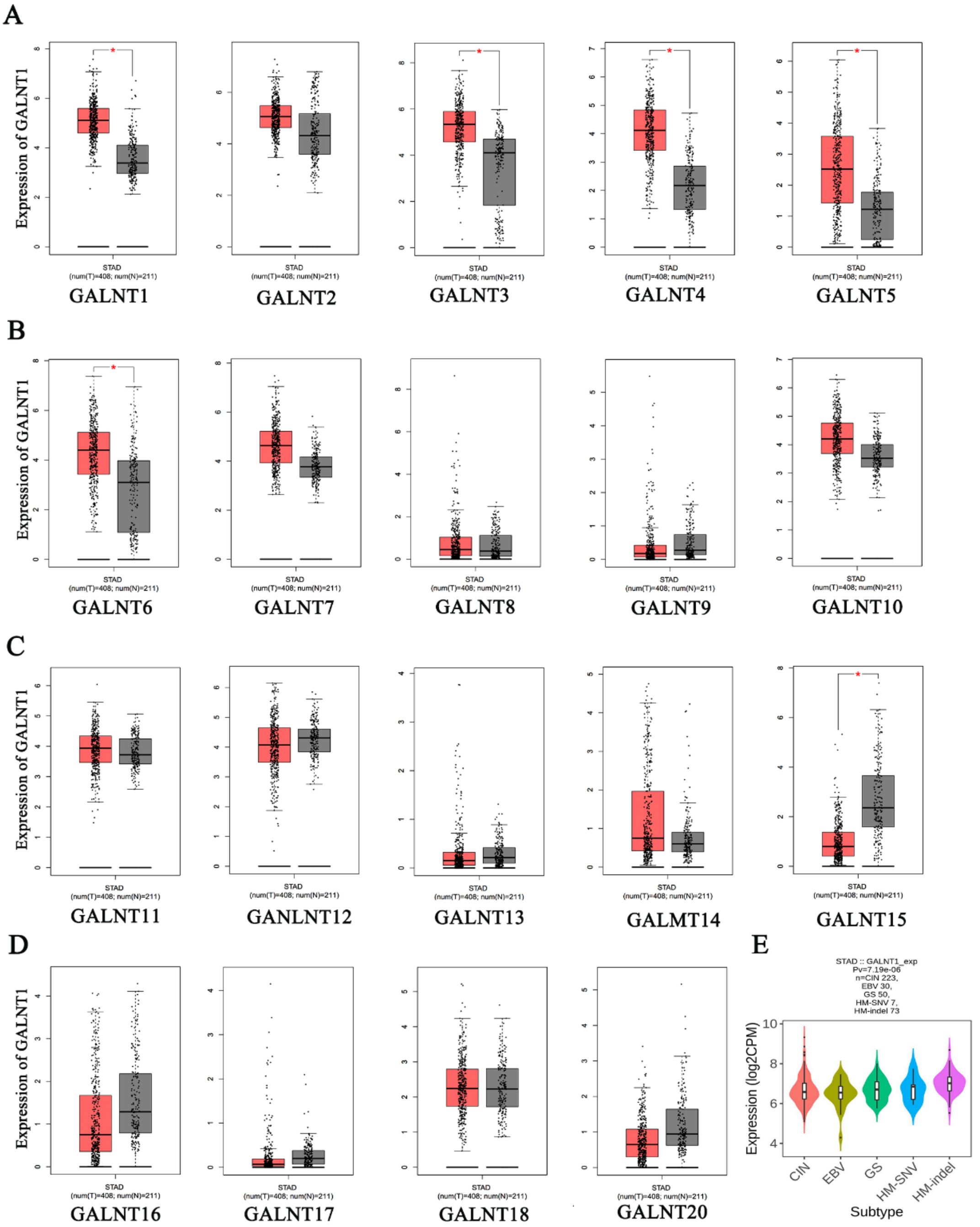

Supplementary figure S1: (**A–D**) Analysis of *GALNT*s expression in gastric cancer using GEPIA database. **E**, Analysis of GALNTs expression in gastric cancer each molecular subtype.

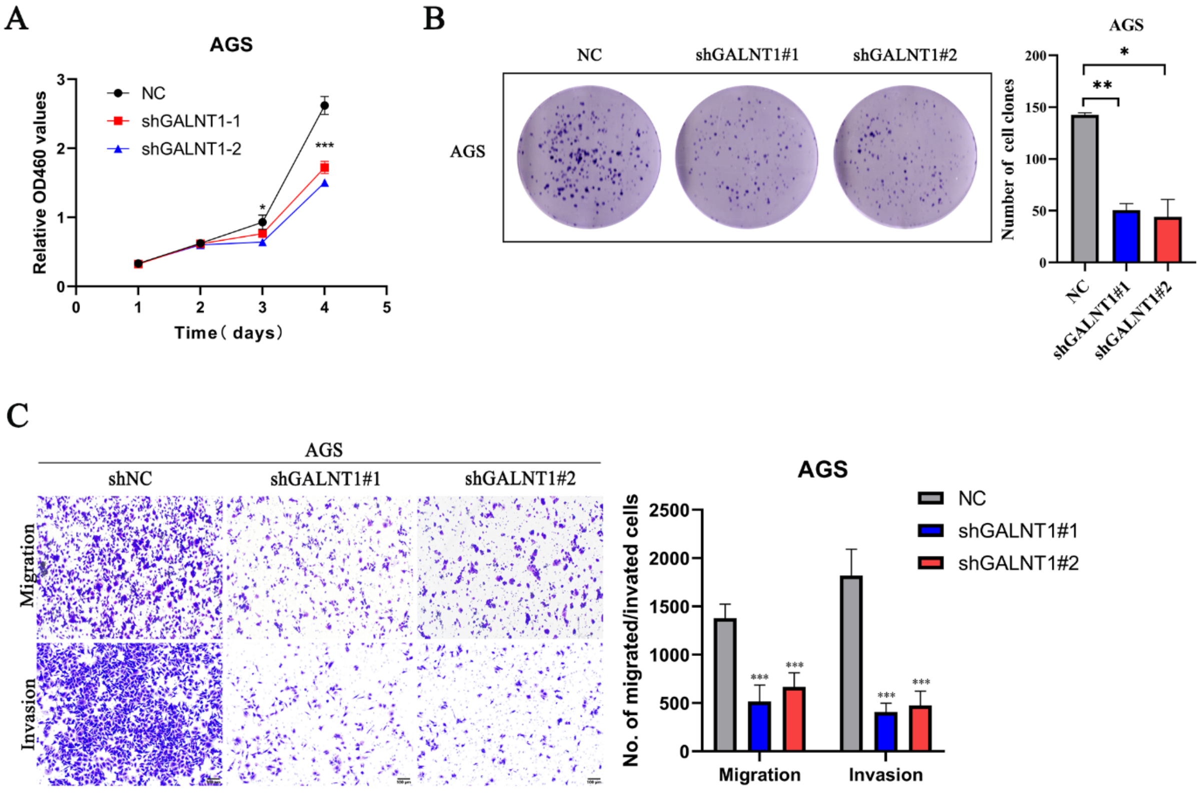

Supplementary figure S2: GALNT1 promotes the growth and metastatic ability of AGS cells and modifies O-glycosylation of CD44. **A**–**B**, AGS cells' growth abilities after *GALNT1* knockdown were determined by CCK8 assay (**A**) and colony formation assay (**B**). **C**, *GALNT1* knockdown decreased the abilities of migration and invasion of AGS cells.

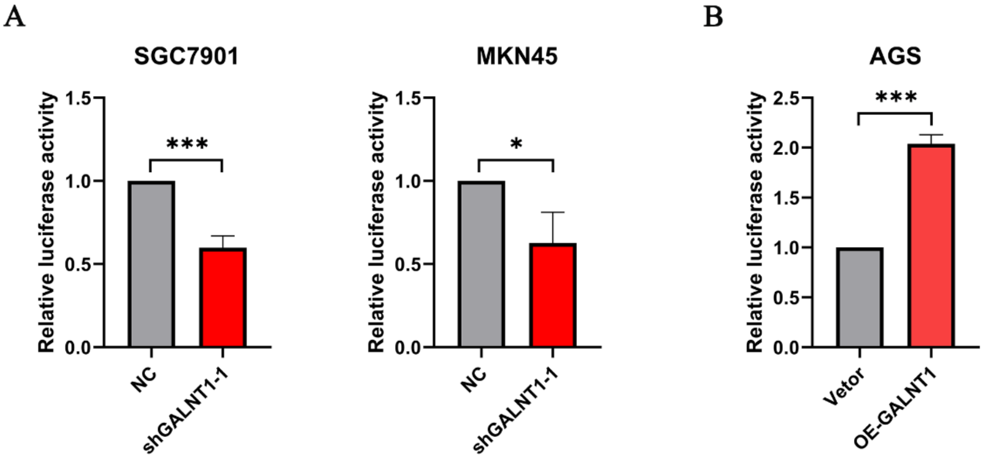

Supplementary figure S3: TOP luciferase reporter assays were measured in GALNT1-silenced (**A**) and -overexpressing cells (**B**). Normalization was based on internal Renilla luciferase activity.

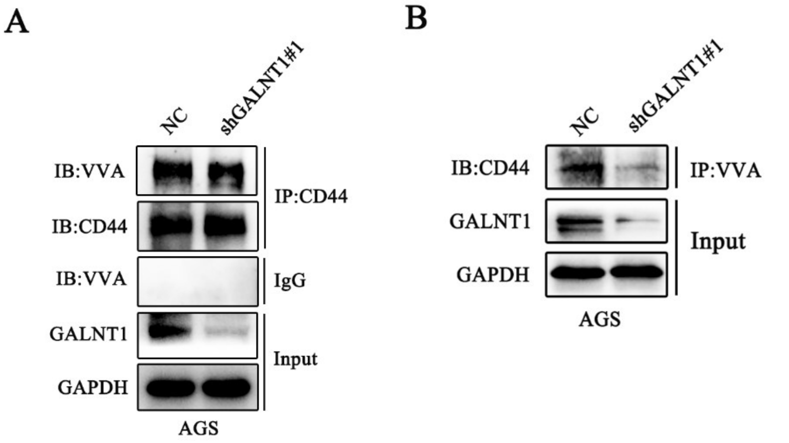

Supplementary figure S4: *GALNT1* knockdown decreased the O-glycosylation levels of CD44 by co-immunoprecipitation in AGS cells (**A-B**).

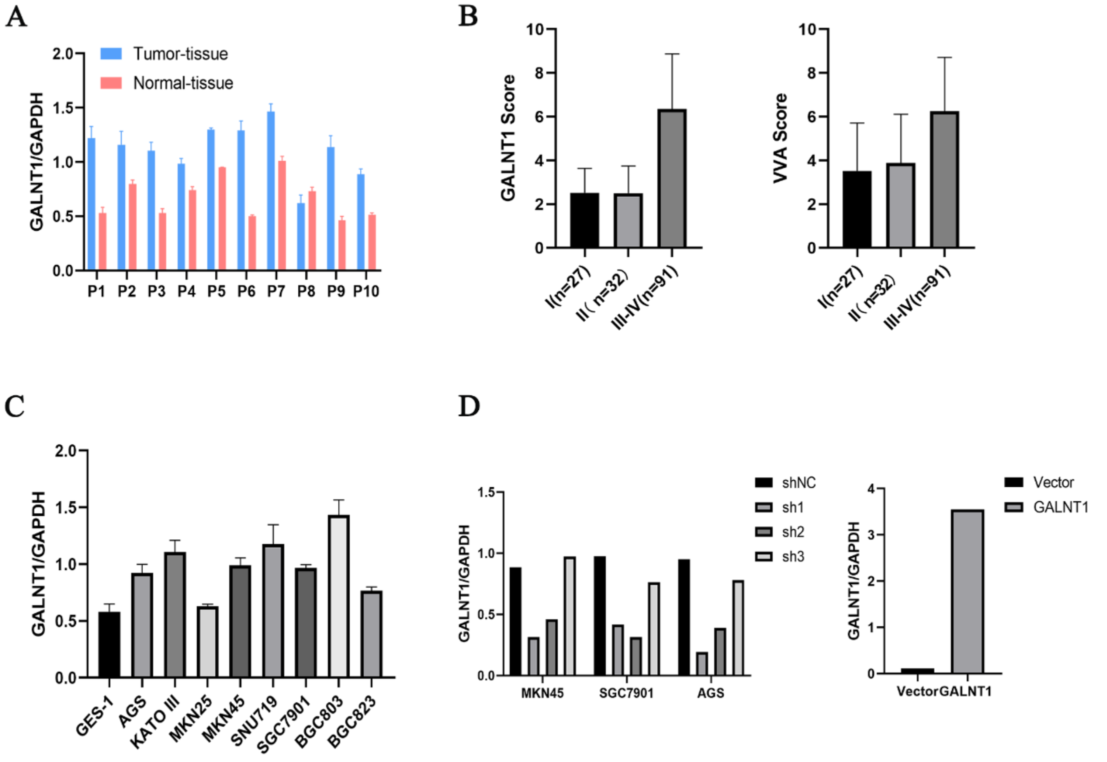

Supplementary figure S5: (A) The quantification of *GALNT1* levels in paired primary tumors and adjacent normal tissues. **(B)** Statistical analysis of *GALNT1* staining at different clinical stages. **(C)** The quantification of *GALNT1* levels in gastric cancer cell lines. **(D)** The quantification of the silencing efficiency of GALNT1 in MKN45, AGS, and SGC7901 cells as well as the overexpression of GALNT1 in AGS cells.

Table S1. The target sequence of sh*GALNT1* and shCD44

Gene	Target sequence
sh*GALNT1*-1	5′− GCTTGGATGTTTCCAAACTTA −3’
sh*GALNT1*-2	5′− ATTGATCAGAGCTAGATTAAA −3’
sh*GALNT1*-3	5′− GACGTGAAACTGCATAGTAAT −3’
ShCD44-1	5′− GGCGCAGATCGATTTGAATAT−3'
shCD44-2	5′− CCAGTATGACACATATTGCTT−3'
shCD44-3	5′−CCATTCAAATCCGGAAGTGCT−3'

Table S2. Sequences of primers used in this study for qRT-PCR.

Gene	Sequence
GALNT1	F:5′- GTGAGATGATTGCACTCAACAGA -3′
	R:5′- ACTATGGACAGTTCGCAGAAGT -3′
GAPDH	F:5′- CGGAGTCAACGGATTTGGTCGT -3′
	R:5′- TCTCAGCCTTGACGGTGCCA -3′
CD44	F:5′- TCCTCACATCCAACACCTCC-3′
	R:5′- GCTGCTCACGTCATCATCAG -3′

Figuer 2B P1-P4 P5-P8

GALNT1

GAPDH

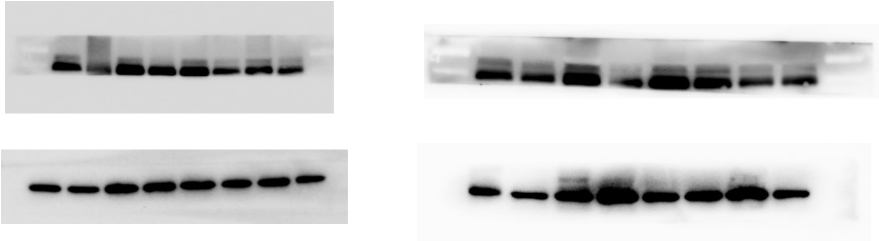

other P9-P10

GALNT1

GAPDH

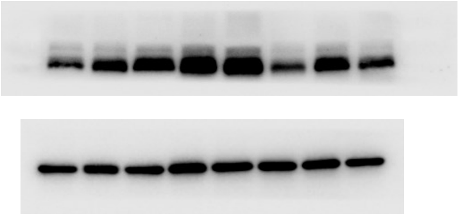

Figuer 3A

GALNT1

GAPDH

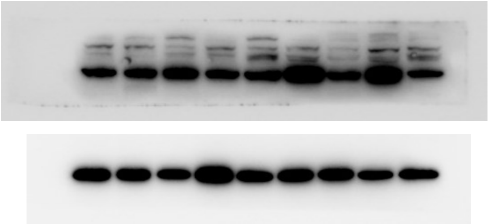

SGC7901

Figuer 3B MKN45

GALNT1

GAPDH

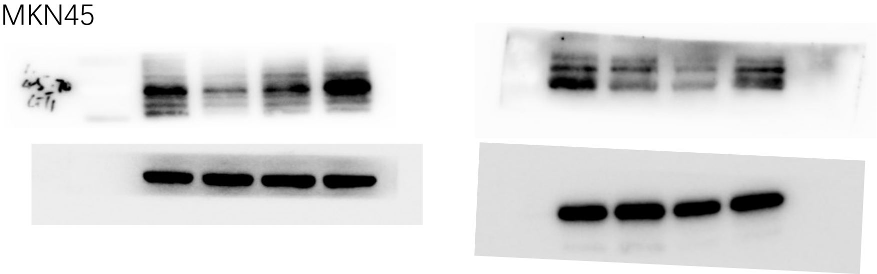

AGS AGS-OE

GALNT1

GAPDH

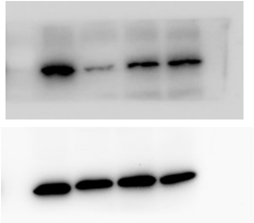

GALNT1

GAPDH

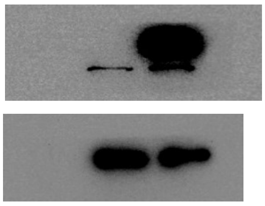

Figure 5C-F SGC7901 AGS

β-catein

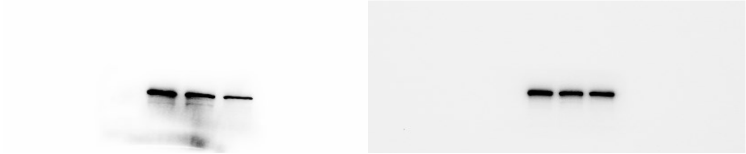

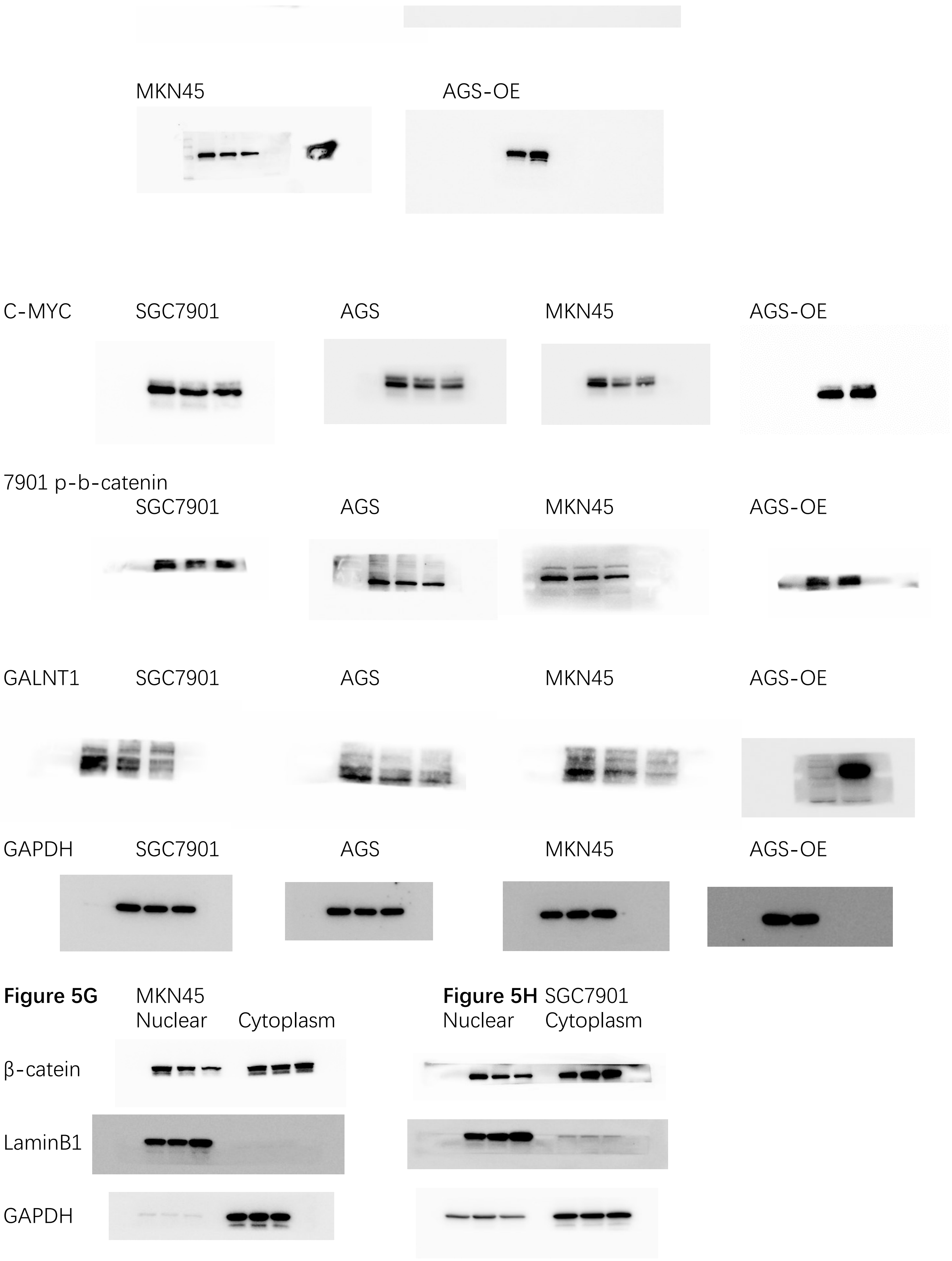
MKN45
AGS-OE
C-MYC
SGC7901
AGS
MKN45
AGS-OE
7901 p-b-catenin
SGC7901
AGS
MKN45
AGS-OE
GALNT1
SGC7901
AGS
MKN45
AGS-OE
GAPDH
SGC7901
AGS
MKN45
AGS-OE
Figure 5G
MKN45
Nuclear
Cytoplasm
β-catein
LaminB1
GAPDH
Figure 5H
SGC7901
Nuclear
Cytoplasm

Figure 5I AGS

Nuclear Cytoplasm

β-catein

LaminB1

GAPDH

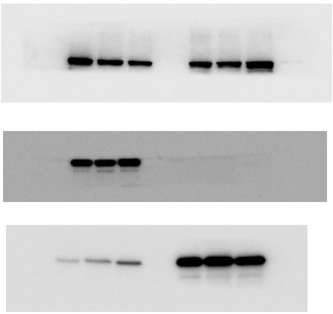

Figure 5J AGS-OE

Nuclear Cytoplasm

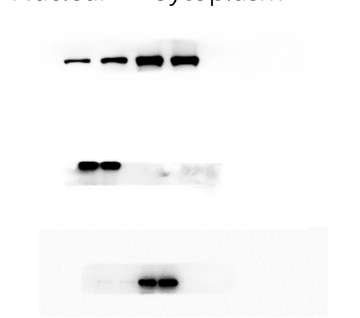

Figure 6A MKN45

INPUT IP:CD44 IP:CD44

CD44

GALNT1

GAPDH

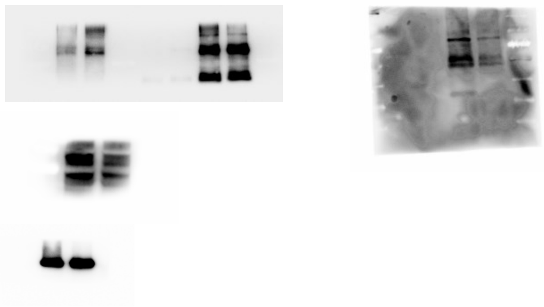

IB:VVA

Figure 6B SGC7901

INPUT IP:CD44 IP:CD44

CD44

GALNT1

GAPDH

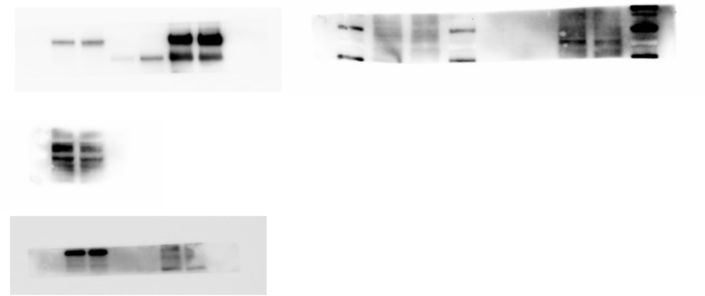

IB:VVA

Figure 6C AGS-OE

INPUT IP:CD44 IP:CD44

CD44

GALNT1

GAPDH

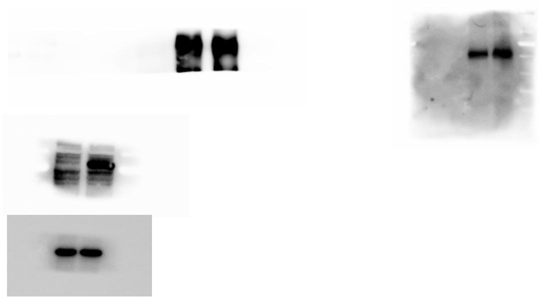

IB:VVA

Figure 6D MKN45

IP:VVA

IB:CD44

GALNT1

GAPDH

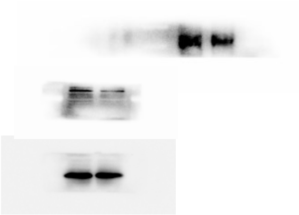

Figure 6E SGC7901

IP:VVA

IB:CD44

GALNT1

GAPDH

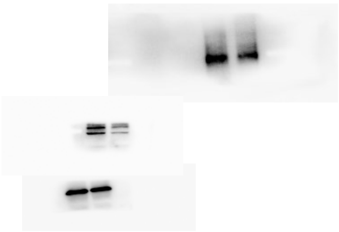

Figure 6F AGS-OE

IP:VVA

IB:CD44

GALNT1

GAPDH

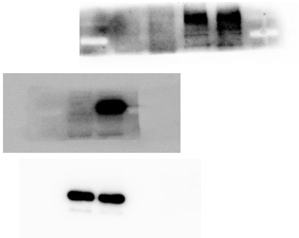

Figure 6G

AGS-OE-shCD44 other

CD44

GAPDH

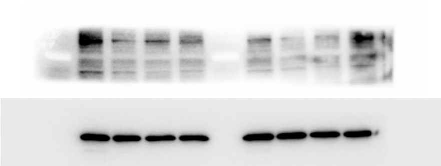

Figure 6K AGS-OE-shCD44

Nuclear cytoplasm

β-catenin

LaminB1

GAPDH

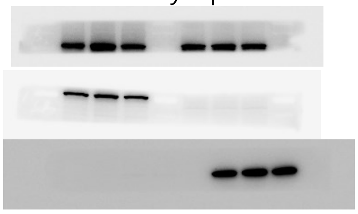

Figure S4A AGS

INPUT IP:CD44 IP:CD44

CD44 IB:VVA

GALNT1

GAPDH

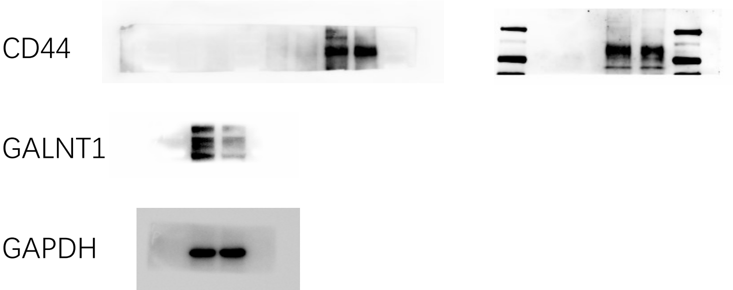

Figure S4B AGS

IP:VVA

IB:CD44

GALNT1

GAPDH

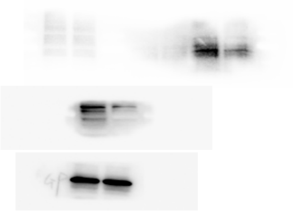

Supplement: Supplementary file 1 — Supplementary figures and tables. [file ijbsv18p6068s1.pdf]
